# Bu-Shen-Tian-Jing Formula alleviates oxidative-inflammatory stress in granulosa cells of polycystic ovary syndrome through AGEs-RAGE/NOX4/NF-κB pathway

**DOI:** 10.1186/s13020-026-01333-z

**Published:** 2026-01-23

**Authors:** Qing Zhang, Jun Ren, Jiayu Ye, Fan Chen, Fuying Xiang, Biwei Shi, Zhishan Zhou, Jinhong Zhou, Fangfang Wang, Fan Qu

**Affiliations:** 1 Zhejiang Hospital, Hangzhou, 310000 Zhejiang China; 2 Women’s Hospital, School of Medicine, Zhejiang University, 1 Xueshi Road, Hangzhou, 310006 Zhejiang China; 3 Zhejiang Chinese Medical University, Hangzhou, 310053 Zhejiang China; 4 Zhejiang Provincial Key Laboratory of Traditional, Chinese Medicine for Reproductive Health Research, Hangzhou, 310006 Zhejiang China

**Keywords:** Polycystic ovary syndrome, Bu-Shen-Tian-Jing Formula, AGEs/RAGE pathway, NADPH oxidase, NF-κB

## Abstract

**Background:**

Polycystic ovary syndrome (PCOS) is a prevalent reproductive endocrine disorder. The traditional Chinese medicine Bu-Shen-Tian-Jing Formula (BSTJF) has demonstrated efficacy in ameliorating PCOS-related pathologies, however its therapeutic mechanisms remain incompletely understood. This study aimed to investigate the pharmacological mechanisms by which BSTJF improves ovarian microenvironment in PCOS.

**Methods:**

BSTJF-containing serum was applied to PCOS granulosa cells (GCs) in vitro for cellular functional assays and transcriptomic sequencing, combined with mass spectrometry-based identification of bioactive components. Network pharmacology and molecular docking were employed to predict multi-target mechanisms of BSTJF against PCOS. In vivo validation utilized an androgen-induced PCOS mouse model divided into five groups: control, PCOS, low-dose BSTJF, high-dose BSTJF, and FPS-ZM1 (RAGE inhibitor). The estrous cyclicity, glucose tolerance, reproductive hormones, ovarian morphology, and granulosa cell apoptosis of mice were detected. Serum inflammatory cytokines and biomarkers of oxidative stress in ovarian GCs were measured. Untargeted metabolomics was employed for comprehensive metabolic profiling in the serum of mice. Molecular analyses included AGEs-RAGE-NOX4 axis expression in GCs, paralleled by p38 MAPK phosphorylation kinetics and NF-κB p65 nuclear translocation dynamics.

**Results:**

Transcriptomic analysis identified differentially expressed genes with significant enrichment in the AGEs-RAGE signaling pathway, revealing oxidative-inflammatory regulatory hubs (NOX4, SOD3, GPX2; TNF, TLR7, CCR2). Network pharmacology provided supports of BSTJF’s multi-target engagement, demonstrating high-affinity interactions between its bioactive components and core targets. In vivo, BSTJF mirrored the RAGE inhibitor FPS-ZM1’s efficacy by ameliorating PCOS phenotypes through reducing GC apoptosis, attenuating AGEs accumulation, inflammatory cytokines and state of oxidative stress, normalizing carbohydrate metabolism and lipid homeostasis, and inhibiting AGEs-RAGE-NOX4 axis activation and NF-κB nuclear translocation in ovarian GCs.

**Conclusion:**

Our study indicated that BSTJF could ameliorate oxidative-inflammatory stress in ovarian GCs of PCOS through AGEs-RAGE/NOX4/NF-κB pathway.

**Supplementary Information:**

The online version contains supplementary material available at 10.1186/s13020-026-01333-z.

## Introduction

Polycystic ovary syndrome (PCOS) represents a prevalent reproductive endocrine disorder affecting women, characterized by oligomenorrhea, hyperandrogenism, and polycystic ovarian morphology [1]. The clinical manifestations of PCOS exhibit significant heterogeneity, frequently accompanied by systemic alterations such as neuroendocrine dysregulation, insulin resistance, dyslipidemia, and chronic low-grade inflammation, collectively exacerbating metabolic, reproductive and genetic risks [2–4]. For PCOS patients with fertility aspirations, weight management and lifestyle modification serve as first-line interventions, supplemented by insulin sensitizers, ovulation induction agents, and assisted reproductive technologies [5]. While these therapeutic approaches demonstrate partial efficacy in addressing ovulatory dysfunction, clinical challenges persist including impaired follicular recruitment, suboptimal conception rates, elevated miscarriage incidence, and gestational complications [6–8]. Alterations in the ovarian microenvironment, encompassing a multifaceted interaction among oxidative stress, inflammatory cascades, and dysregulated secretion of regulatory factors, have been implicated in the suboptimal reproductive outcomes associated with PCOS [9–11]. This pathophysiological interconnection underscores for more effective therapeutic options capable of ameliorating ovarian microenvironmental dynamics in PCOS populations.

Traditional Chinese medicine (TCM) formulations exhibit distinctive advantages in PCOS management. Guided by holistic principles, TCM prescriptions synergistically correct both reproductive impairments and metabolic disturbances through multi-target pharmacological interactions [12–14]. The Bu-Shen-Tian-Jing formula (BSTJF), a Chinese herbal medicine developed by our research team for PCOS-related infertility treatment, operates on the foundational therapeutic principle of renal reinforcement and essence replenishment. Clinical validation confirms BSTJF’s therapeutic efficacy, with a retrospective study documenting its capacity to enhance assisted reproductive outcomes in PCOS patients [15]. Experimental pharmacological studies further demonstrate that BSTJF intervention in PCOS rodent models effectively ameliorates follicular developmental arrest [16] and improves neurobehavioral profiles in offspring [17]. These substantiate BSTJF’s positive regulatory actions on PCOS reproductive pathophysiology, providing mechanistic direction for further research into its therapeutic mechanisms.

Advanced glycation end-products (AGEs), toxic compounds formed through non-enzymatic glycation and oxidative modification of proteins/lipids, undergo pathological accumulation accelerated by aging, hyperglycemia, obesity, insulin resistance, and dietary intake of glycotoxins [18]. Emerging evidence has delineated the pathophysiological involvement of AGEs in PCOS pathogenesis, with elevated circulating AGEs exhibiting significant correlations with hyperandrogenemia, adiposity, and metabolic dysfunction in clinical cohorts [19–22]. The AGEs-RAGE (receptor for AGEs) axis activates NADPH oxidase upon ligand-receptor binding, propagating downstream inflammatory signaling cascades through nuclear factor-κB (NF-κB)-mediated transcriptional activation of proinflammatory cytokines, while concurrently amplifying intracellular reactive oxygen species (ROS) generation [23]. Excessive ROS triggers oxidative stress via p38 mitogen-activated protein kinase (p38-MAPK) activation and establishes a positive feedback loop by upregulating RAGE expression, thereby perpetuating a self-reinforcing cycle of oxidant-inflammatory damage [24, 25]. Notably, NADPH oxidase serves as the principal enzymatic source of intracellular ROS [26], with its activity directly modulated by the AGEs-RAGE system [18]. Among NADPH oxidase isoforms, NOX4 demonstrates ubiquitous expression across renal, vascular, and neural tissues. Previous investigations have identified ovarian granulosa cell expression of NOX4 [27, 28], with experimental evidence confirming its mechanistic role in mediating oxidative damage and apoptotic signaling in PCOS rodent model [29].

**Fig. 1:**
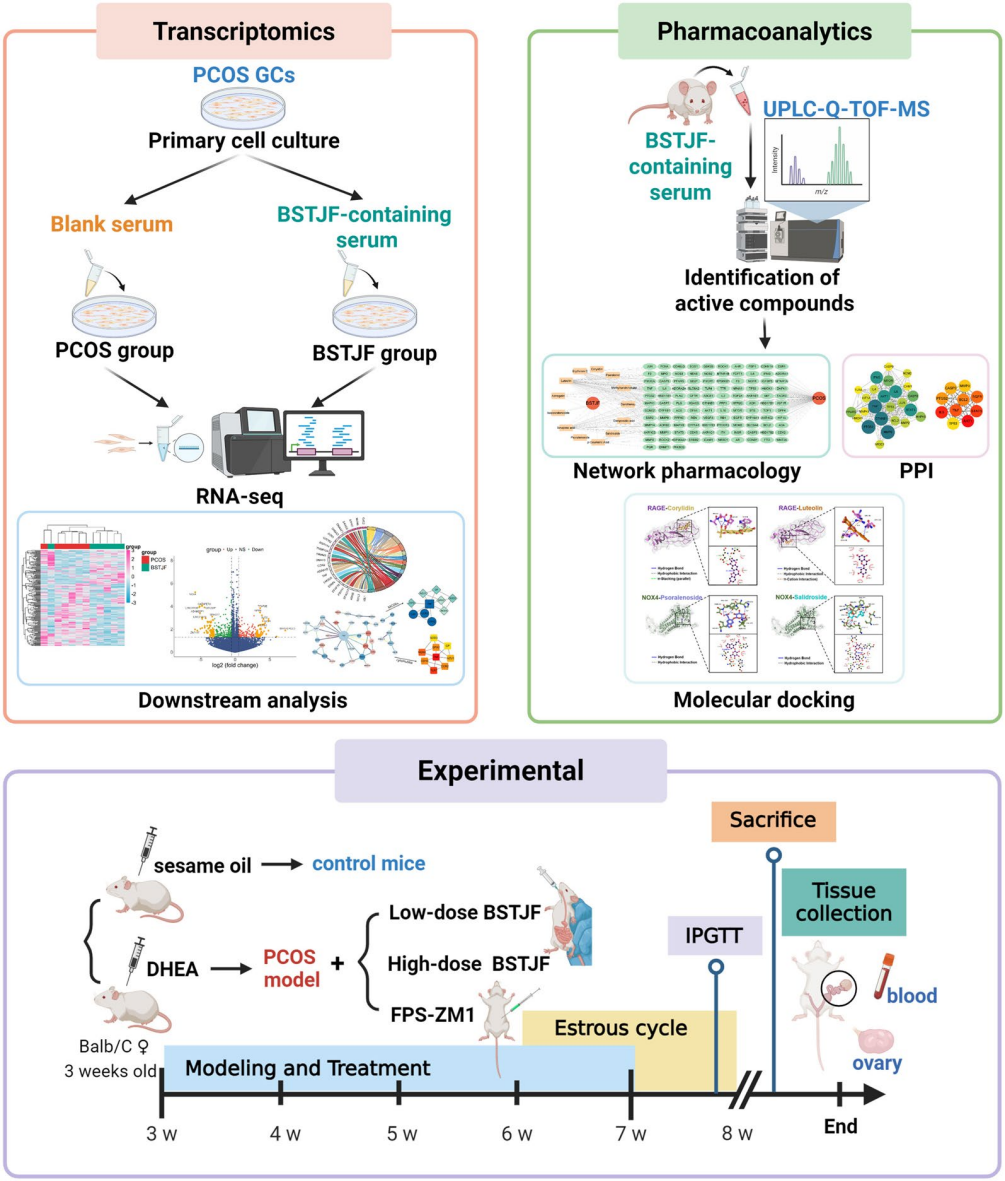
The workflow diagram for this study. Initially, transcriptomic profiling of BSTJF-treated granulosa cells (GCs) using drug-containing serum was integrated with mass spectrometry-based characterization of serum components and network pharmacology to systematically identify bioactive compounds and their therapeutic targets/pathways. Subsequent in vivo investigations demonstrated BSTJF’s efficacy and therapeutic mechanisms in PCOS mouse models

Building upon these mechanistic insights, we postulate that AGEs-RAGE axis dysregulation, through aberrant NOX4 and NF-κB signaling, drives oxidative stress and inflammation in ovarian granulosa cells (GCs) of PCOS. While the therapeutic benefits of BSTJF in PCOS are increasingly recognized, its molecular targets remain incompletely elucidated. Our prior mechanistic study elucidated BSTJF’s modulation on PCOS pathogenesis via Sirtuin 3-driven mitochondrial oxidative stress amelioration [16]. Moreover, key components of the BSTJF formula have been previously reported to possess potent anti-oxidant and anti-inflammatory properties [30], particularly through the modulation of AGEs-RAGE pathway in models of diabetes or metabolic disorders [31]. Given the prominence of the AGEs-RAGE/NOX4/NF-κB pathway in driving PCOS pathogenesis, as outlined above, we hypothesized that BSTJF might ameliorate PCOS by modulating this oxidative-inflammatory pathway. To test this hypothesis, in our study, transcriptomic profiling of BSTJF-treated GCs using drug-containing serum was integrated with mass spectrometry-based characterization of serum components and network pharmacology, subsequent in vivo investigations demonstrated BSTJF’s efficacy and elucidated its molecular mechanisms through AGEs-RAGE axis modulation. The whole flow chart of the study is summarized in Fig. 1.

## Materials and methods

### Preparation of BSTJF and drug-containing serum

BSTJF were purchased from Huadong Medicine Co., Ltd (Hangzhou, China). BSTJF consists of seven herbs: *Rehmanniae* Radix Praeparata (Shu Di Huang, 20 g), *Ligustri* Lucidi Fructus (Nv Zhen Zi, 20 g), *Rubi* Fructus (Fu Pen Zi, 10 g), *Cuscutae* Semen (Tu Si Zi, 15 g), *Psoraleae* Fructus (Bu Gu Zhi, 15 g), *Astragali* Radix (Huang Qi, 10 g), and *Salviae* Miltiorrhizae Radix (Dan Shen, 10 g). Herbs were hydrated in distilled water, decocted at 100 °C for 90 min, and filtered. The supernatant was concentrated via rotary evaporation and stored at 4 °C for subsequent experiments.

Twenty healthy female Sprague–Dawley rats (200 ± 20 g, SPF-grade), were obtained from the Laboratory Animal Research Center of Zhejiang Chinese Medical University (Ethics Approval No.: IACUC-20230710-08). Animals were housed under controlled conditions (25 °C, 55% humidity, 12 h light/dark cycle) with free access to food and water. Rats were randomly divided into two groups including BSTJF-treated group and control group. Rats in BSTJF-treated group were treated intragastrically with BSTJF at a dose of 3.4 g/kg/day to obtain BSTJF-containing serum, while the control group received sterile water intragastrically to obtain blank serum. Treatments were administered at 8: 00 am and 8: 00 pm daily for 4 consecutive days. Abdominal aortic blood samples were withdrawn under sterile conditions 1 h after the last dose. The blood samples were aseptically packed and centrifuged at 3,000 rpm for 15 min. Then, the serum was inactivated using a water bath at 56 °C for 30 min, sterilized using a 0.22 μm filter membrane, and stored at − 80 °C for future use.

### UPLC-Q-TOF-MS of BSTJF-containing serum

The chemical constituents in BSTJF-containing serum (i.e., blood-absorbed components) were analyzed through ultra-high performance liquid chromatography coupled with quadrupole time-of-flight mass spectrometry (UPLC-Q-TOF MS). The separation was performed on a CORTECS C18 column (4.6 × 100 mm, 2.7 μm) with mobile phase A (0.1% formic acid aqueous solution) and B (0.1% formic acid in acetonitrile) under gradient elution: 0 min, 2% B; 5 min, 5% B; 12 min, 12% B; 18 min, 18% B; 28 min, 30% B; 32 min, 36% B; 36 min, 42% B; 40 min, 70% B; 45 min, 70% B. The flow rate was maintained at 0.7 mL/min with column temperature set at 30 °C and injection volume of 10 μL. For mass spectrometric detection, an electrospray ionization source was operated in negative ion mode with the following parameters: nebulizing gas (N2): 30 psi Curtain gas; gas 1 & 2: 50 psi each; ion spray voltage: − 4.5 kV; collision energy: 10 V; declustering potential: 100 V; turbo electrospray temperature: 550 °C; Full scan range: m/z 100–2000. Data acquisition was conducted using MassLynx V4.1 software. The isotopic abundance patterns and MS/MS spectra were systematically analyzed through Progenesis QI software. High-resolution exact masses were employed to deduce molecular formulas for each chromatographic peak. Putative identifications were further confirmed by comparing retention times and MS/MS fragmentation patterns with reference standards. The identification of the bioactive components was qualitative, based on comparing their retention times and accurate mass spectra with those of available reference standards or database records.

### Cell culture and treatment

Ovarian GCs were collected from follicular aspirates of PCOS patients undergoing controlled ovarian stimulation for in vitro fertilization (IVF) at the Reproductive Medicine Center of Women’s Hospital, School of Medicine, Zhejiang University, Hangzhou, China. The hospital’s Institutional Review Board approved the study protocol and informed consents were obtained from all participants (Approval No.:20180129). PCOS was diagnosed according to the 2003 Rotterdam Consensus. GC isolation was performed via density gradient centrifugation from pooled follicular fluids, as previously optimized [32]. Primary GCs were cultured in DMEM/F12 medium (Biological Industries, Israel), supplemented with 10% foetal bovine serum (FBS) (Biological Industries, Israel), and 100 U/mL of penicillin/streptomycin (Biological Industries, Israel) at 37 °C in a humidified atmosphere, with 5% CO_2_. For each sample, GCs were seeded into 6-well plates at a density of 1 × 10^6^ cells/well and were devided into two groups. After 6 h of serum starvation, GCs were treated with 20% BSTJF-containing serum for 24 h as the BSTJF group, while cells in other group received an equivalent volume of blank serum for the same duration. The 20% concentration of BSTJF-containing serum used in cell experiments was selected based on pharmacokinetic equivalence to the clinical dose. This concentration was further verified to have no cytotoxic effect in our preliminary CCK-8 assay.

### Cellular functional assays

Apoptosis was evaluated by flow cytometry using an Annexin V-FITC/PI Apoptosis Detection Kit (Beyotime Biotechnology), with early and late apoptotic populations quantified based on fluorescence staining. DNA fragmentation was further assessed by a TUNEL Apoptosis Detection Kit (Beyotime Biotechnology), wherein fixed and permeabilized GCs were incubated with TUNEL reaction mix and counterstained with DAPI for fluorescence microscopic quantification. Intracellular ROS levels were measured using the DCFH-DA probe (Beyotime Biotechnology). After incubation and washing, cells were stained with Hoechst 33,342 for nuclear labeling and visualized under a confocal microscope (ZEISS, Germany) for image acquisition. Inflammatory cytokines (IL-1β, IL-6, IL-10, TNF-α) and AGEs was quantified from culture supernatants using commercial ELISA kits according to manufacturer protocols, with absorbance read at 450 nm.

### Transcriptomic profiling

RNA sequencing was performed on six GC samples per group following intervention with BSTJF-containing serum or blank serum, respectively. Total RNA of GCs was isolated and purified using TRIzol reagent (Invitrogen, USA). Total RNA was extracted using TRIzol™ (Invitrogen, USA), with purity assessed by NanoDrop ND-1000 (OD260/280 ≥ 1.8) and integrity verified via Bioanalyzer 2100 (RIN ≥ 7.0, Agilent Technologies). Stranded mRNA-seq libraries were constructed from qualified samples (RNA concentration > 50 ng/μL, total RNA ≥ 1 μg) and sequenced (PE150) on an Illumina NovaSeq 6000 platform (LC-Bio Technology CO., Ltd., Hangzhou, China).

Raw reads were preprocessed using Cutadapt software to remove adapter sequences and low-quality bases. Clean reads were aligned to the human reference genome (GRCh38) using HISAT2 software, followed by transcript assembly and quantification via StringTie and GffCompare softwares with fragment per kilobase of transcript per million mapped reads (FPKM) normalization. Differentially expressed genes (DEGs) were identified using R package-DESeq2 with thresholds of |log2FC|> 1 and *P* < 0.05. For functional enrichment, clusterProfiler was employed to perform Gene Ontology (GO) and Kyoto Encyclopedia of Genes and Genomes (KEGG) pathway analyses. Gene Set Enrichment Analysis (GSEA) was conducted via the fgsea package, ranking genes by log2 fold change and testing against the Molecular Signatures Database (MSigDB) hallmark gene sets. Enrichment results were visualized using ggplot2, with redundant terms collapsed by semantic similarity (SimplifyEnrichment, v1.10.0). All analyses utilized the org.Hs.eg.db for gene identifier mapping.

### Network pharmacology

The putative bioactive components in BSTJF-containing serum were subjected to target prediction using TCMSP, SwissTargetPrediction, STITCH, and PharmMapper databases, while PCOS-related disease targets were retrieved from Genecards, OMIM, DisGenet, and DrugBank. The intersection between compound-predicted targets and disease-associated targets was identified via an online Venn diagram tool [33] to obtain shared therapeutic targets for BSTJF against PCOS. These overlapping targets were imported into the STRING database to generate a protein–protein interaction (PPI) network, which was subsequently visualized and refined using Cytoscape (v3.9.0) to construct a compound-disease-target network. Functional enrichment analysis of the shared targets was performed through GO and KEGG pathway assessments using the R package clusterProfiler, with results visualized via enrichplot and ggplot2 packages to identify pivotal signaling pathways underlying BSTJF’s therapeutic effects on PCOS.

### Molecular docking

The structural formulas of bioactive components in BSTJF-containing serum were retrieved in SDF format from the PubChem database. These compounds were energy-minimized using Chem3D software to generate optimized 3D conformations, which were subsequently exported as MOL2 files. The crystal structures of core protein domains were acquired from the Protein Data Bank (PDB) and preprocessed in PyMOL through removal of water molecules, phosphate groups, and heteroatoms. Molecular docking simulations were performed using AutoDockTools 1.5.7 and Discovery Studio 2021, with Lamarckian genetic algorithm parameters set to 100 runs and 25 million energy evaluations. Binding conformations with the lowest Gibbs free energy (ΔG < − 5 kcal/mol) were prioritized for analysis. Binding affinity calculations and intermolecular interaction visualizations were conducted to predict the regulatory effects of bioactive components on core therapeutic targets. Our approach is consistent with similar in silico docking strategies employed in recent phytochemical studies [34].

### PCOS mouse model and BSTJF intervention

Animal experiment was performed according to the Care and Use of Laboratory Animals protocol of National Research Council of China, and was approved by the Ethics Committee of Zhejiang Chinese Medical University on March 18th, 2024 (Approval No.: IACUC-20240318-05). Female pre-puberty C57BL/6 mice (21 days old) were obtained from the Laboratory Animal Research Center, Zhejiang Chinese Medical University, Hangzhou, China. They were randomly allocated into five groups (n = 8/group): Control, PCOS model, BSTJF low-dose (BSTJF-L), BSTJF high-dose (BSTJF-H), and FPS-ZM1 (a RAGE inhibitor) groups. Following established protocols (ovarian RAGE upregulation was reported in this androgen-induced PCOS model) [35, 36], the PCOS model was induced by daily subcutaneous injections of dehydroepiandrosterone (DHEA) for 21 consecutive days. DHEA was administered at a dose of 6 mg/100 g body weight and dissolved in 98% sesame oil (Macklin, S905724). Control mice received equivalent volumes of sesame oil via the same route. The clinical recommended dosage of BSTJF for a 60 kg adult is 100 g/d, and the equivalent dose for mice, based on body surface area, is 10 times (17 g/kg/day) that of humans. Thus, BSTJF-L and BSTJF-H groups were administered BSTJF via oral gavage twice daily at 1.7 g/mL and 3.4 g/mL doses, respectively (1 mL/100 g body weight per dose), while other groups received equal volumes of distilled water for 4 weeks. Concurrently, the FPS-ZM1 group received daily intraperitoneal injections of FPS-ZM1 (0.1 mg/100 g body weight in DMSO diluted with saline), whereas other groups were injected with equivalent volumes of diluted DMSO vehicle for 4 weeks.

### Vaginal smears

At 6 weeks of age, female mice underwent daily vaginal cytology for 14 consecutive days to assess estrous cyclicity. Vaginal lavage was performed using a saline-moistened cotton swab gently rotated against the vaginal wall. Lavage fluid was smeared onto glass slides, air-dried, fixed in methanol, and stained with hematoxylin and eosin (H&E). Estrous phases (proestrus, estrus, metestrus, diestrus) were classified microscopically based on dominant epithelial cell morphology (nucleated, cornified, or leukocytic) as a previous study described [37].

### Glucose tolerance

Following BSTJF intervention, mice underwent a 12-h overnight fast prior to intraperitoneal glucose tolerance testing (IPGTT). Fasting blood glucose levels were quantified via tail-vein sampling using an Accu-Chek^®^ glucometer with standardized test strips (Roche Diagnostics). After a baseline (fasted) measurement, the mouse were injected intraperitoneally with 20% glucose solution in a volume of 1 mL/100 g, and the glucose levels were measured at 30, 90, and 120 min.

### Ovarian morphometry

When mouse were eight weeks old, bilateral ovaries were harvested from euthanized mice. Tissues were perfusion-fixed in 4% paraformaldehyde, dehydrated through graded ethanol series, paraffin-embedded, and sectioned coronally at 4 µm thickness. Serial sections were stained with H&E for histopathological evaluation. Follicles at different developmental stages (primordial, primary, secondary, antral) and corpora lutea were counted.

### Enzyme-linked immunosorbent assay (ELISA)

Blood samples were collected from the sacrificed mouse. Serum concentrations of free testosterone, luteinizing hormone (LH), follicle-stimulating hormone (FSH), interleukin-10 (IL-10), interleukin-6 (IL-6), tumor necrosis factor-alpha (TNF-α), interleukin-1 beta (IL-1β) and AGEs were quantified using commercial ELISA kits (Jingmei Biotechnology) following manufacturer protocols. Briefly, blood samples were collected via retro-orbital bleeding, allowed to clot at room temperature for 30 min, and centrifuged at 3000 rpm for 15 min at 4 °C to isolate serum. All samples were stored at − 80 °C until analysis. For each target analyte, 96-well plates pre-coated with specific capture antibodies were incubated with 50 μL of fivefold diluted serum alongside standard curve dilutions in duplicate. Plates were incubated at 37 °C for 1–2 h, followed by sequential additions of biotinylated detection antibodies, streptavidin–horseradish peroxidase (HRP) conjugate, and tetramethylbenzidine (TMB) substrate. Reactions were terminated with 2 N H₂SO₄, and absorbance was measured at 450 nm (630 nm reference) using a microplate reader (Labsystems Multiskan MS). The concentrations of the above analytes in murine serum samples were determined by calculating a linear regression equation derived from the absorbance values of the standards, and subsequently applying this equation to extrapolate sample concentrations.

### Untargeted metabolomic profiling

Eight samples of murine serum per group were used to carry out metabolomic analysis by liquid chromatography-tandem mass spectrometry (LC–MS/MS). Serum samples were thawed on ice, and proteins were precipitated using acetonitrile/methanol (1:4, v/v) containing internal standards. Following centrifugation (12,000 rpm, 10 min, 4 °C), supernatants underwent freeze–thaw purification before LC–MS/MS analysis. Chromatographic separation was performed on a Waters HSS T3 column (2.1 × 100 mm, 1.8 μm) with 0.1% formic acid in water (A) and acetonitrile (B) under a 10-min gradient (5–99% B). Mass spectrometric detection utilized a Q Exactive HF-X system in both positive (3.5 kV) and negative (3.2 kV) ionization modes, employing full-scan MS (m/z 75–1000 at 35,000 resolution) and data-dependent MS/MS (30/40/50 V collision energies). For multivariate analysis, raw data were unit variance-scaled and subjected to unsupervised PCA (principal component analysis) using R/prcomp. Differential metabolites were identified through OPLS-DA (log2-transformed, mean-centered data) using MetaboAnalystR, applying thresholds of VIP > 1 and *P* < 0.05 (Student’s t-test), validated by 200 permutation tests to prevent overfitting. KEGG pathway enrichment was performed by mapping annotated metabolites (KEGG Compound database) to biological pathways via the KEGG Pathway repository. Data visualization was performed using the Metware Cloud, a free online platform for data analysis (https://cloud.metware.cn).

### Assessment of follicular apoptosis

Apoptotic GCs in ovarian follicles were quantified using the TUNEL assay (Roche, #11684817910). Paraffin-embedded ovarian Sects. (4 µm) were deparaffinized, rehydrated, and incubated with proteinase K (20 µg/mL, 37 °C, 30 min). TUNEL reaction mixture was applied to sections following the manufacturer’s protocol, with 4′,6-diamidino-2-phenylindole (DAPI) counterstaining for nuclear visualization. Fluorescence imaging was performed on a laser scanning confocal microscope (LSM 900, Zeiss) using a Cy3 filter (Ex/Em: 550/570 nm) for TUNEL signal and DAPI channel (Ex/Em: 358/461 nm). Three to five random fields per section (n = 3/group) were analyzed using ImageJ (NIH). Apoptotic index was calculated as: TUNEL fluorescence ratio (%) = (integrated density of TUNEL positive signals)/(total nuclear area) × 100%. Non-specific binding was controlled by omitting terminal transferase in negative controls.

### Immunohistochemistry

Ovarian specimens from the unilateral ovary were fixed in 4% paraformaldehyde (PFA), paraffin-embedded, and sectioned at 4 µm thickness. Following deparaffinization, antigen retrieval was performed using citrate buffer, pH 6.0, at 95 °C for 20 min. Sections were sequentially treated with 3% hydrogen peroxide to quench endogenous peroxidase activity and blocked with 5% bovine serum albumin (BSA) for 1 h at room temperature. Tissue sections were then incubated overnight at 4 °C with the following primary antibodies: anti-AGEs (1:200, bs-1158R, Bioss), anti-RAGE (1:300, 16,346-1-AP, Proteintech), and anti-NOX4 (1:2500, 14,347-1-AP, Proteintech). After PBS washes, sections were exposed to horseradish peroxidase (HRP)-conjugated goat anti-rabbit IgG secondary antibody (1: 2000, ab205718, abcam) at 37 °C for 30 min. Chromogenic development was achieved by incubating with 3,3′-diaminobenzidine (DAB) substrate (Biossci) for 10 min, followed by hematoxylin counterstaining (Biossci) for 5 min. Dehydration through graded ethanol series and xylene clearing preceded mounting with neutral resin. Whole-slide digitization was conducted using a 3DHISTECH Pannoramic SCAN II scanner. Quantitative assessment of immunoreactivity was conducted by analyzing staining intensity in five randomly selected fields per section (n = 3/group) using ImageJ software (NIH, USA) with the IHC Profiler plugin.

### Immunofluorescence quantification

Ovarian tissue sections were fixed in 4% paraformaldehyde, blocked with 3% donkey serum (AntGene) at 37 °C for 30 min, and incubated overnight at 4 °C with primary antibodies against FSHR (1: 100, 22,665-1-AP, Proteintech), 4-Hydroxynonenal (1: 200, HY-P81208, MCE), 3-Nitrotyrosine (1: 100, HY-P81216, MCE), 8-OHdG (1: 100, ab48508, Abcam) and NF-κB p65 (1: 100, ab32536, Abcam). After PBS washes, sections were incubated with Alexa Fluor^®^488-highly cross-adsorbed secondary antibodies (1:200, ThermoFisher) at 37 °C for 45 min in the dark, counterstained with DAPI (Solarbio), and mounted with ProLong™ Diamond antifade medium. Fluorescence imaging was performed using an Olympus BX53 microscope and 3DHISTECH Pannoramic SCAN II (20 × whole-slide scanning). For quantification, five random fields per section (n = 4/group) were analyzed in ImageJ (NIH) to assess the integrated density (IntDen) of target proteins.

### Gene expression analysis by RT-qPCR

Total RNA was extracted from human ovarian GCs and mouse ovarian tissues using TRIzol reagent. cDNA was synthesized from 1 μg of total RNA using the Evo M-MLV Reverse Transcription Kit (Accurate Biology). Quantitative PCR was performed using SYBR Green Premix *Pro Taq* HS qPCR Kit (Accurate Biology) on a QuantStudio 5 Real-Time PCR System. The reaction conditions were as follows: initial denaturation at 95 °C for 30 s, followed by 40 cycles of 95 °C for 5 s and 60 °C for 30 s. All primer sequences are listed in Supplementary Table 1. Gene expression levels were normalized to Actb (for mouse) or ACTB (for human) and analyzed using the 2^ − ΔΔCt method.

### Western blot analysis

The murine ovary tissues were homogenized in ice-cold RIPA lysis buffer (Beyotime) supplemented with protease (MCE) and phosphatase inhibitors (MCE). Lysates were centrifuged at 12,000 × g for 15 min at 4 °C, and supernatants were collected for protein quantification using a BCA assay kit (Beyotime). Equal protein aliquots (30 μg/lane) were resolved electrophoretically on 4–12% Bis–Tris gradient gels (FuturePAGE™, ACE Biotechnology) under denaturing conditions with MOPS-SDS buffer (ACE Biotechnology) using constant voltage (130 V) for 60 min. Proteins were transferred onto methanol-activated 0.22 μm PVDF membranes (Millipore) using a semi-dry transfer system (100 V for 80 min). Membranes were blocked with 5% BSA in TBST (20 mM Tris–HCl, 150 mM NaCl, 0.1% Tween 20, pH 7.6) for 2 h at room temperature, followed by incubation with primary antibodies at 4 °C for 16 h: RAGE (1: 2000, 16,346-1-AP, Proteintech), NOX4 (1: 2000, 14,347-1-AP, Proteintech), superoxide dismutase 2 (SOD2) (1: 5000, 24,127-1-AP, Proteintech), NF-κB p65 (1: 2000, 10,745-1-AP, Proteintech), phospho-NF-κB p65 (Ser468) (1: 2000, 82,335-1-RR, Proteintech), IkBα (1: 1000, PTM-6005, PTM BIO), phospho-IKBα (phospho S32/36) (1: 1000, PTM-6401, PTM BIO), mitogen-activated protein kinase 14 (p38 MAPK) (1: 5000, 14,064-1-AP, Proteintech), phospho-p38 MAPK (Thr180/Tyr182) (1: 5000, 28,796-1-AP, Proteintech), β-tubulin (1: 2000, PTM-6414, PTM BIO). After TBST washes (3 × 10 min), membranes were incubated with HRP-conjugated anti-rabbit or anti-mouse secondary antibodies (1: 5000, Proteintech) for 1 h at 25 °C. Chemiluminescent signals were developed using ECL substrate (FDbio) and captured using an ImageQuant LAS 4000mini system (GE Healthcare) with multiple exposure times (10 s to 5 min). Band intensity quantification was performed using ImageJ (NIH) with background subtraction, normalized to β-tubulin as the loading control.

### Statistical analysis

Data are expressed as mean ± standard deviation (SD). Normality was assessed using Shapiro–Wilk tests. Parametric comparisons were conducted as follows: paired t-test for within-group analyses, independent t-test for two-group comparisons, and one-way ANOVA with Tukey’s post hoc correction for multi-group comparisons. Non-normally distributed data were analyzed using the Mann–Whitney *U* test. All analyses were performed in GraphPad Prism (v9.0, GraphPad Software), with statistical significance defined at *P* < 0.05.

## Results

### Transcriptomic profiling reveals BSTJF-driven gene and pathway regulation

**Fig. 2:**
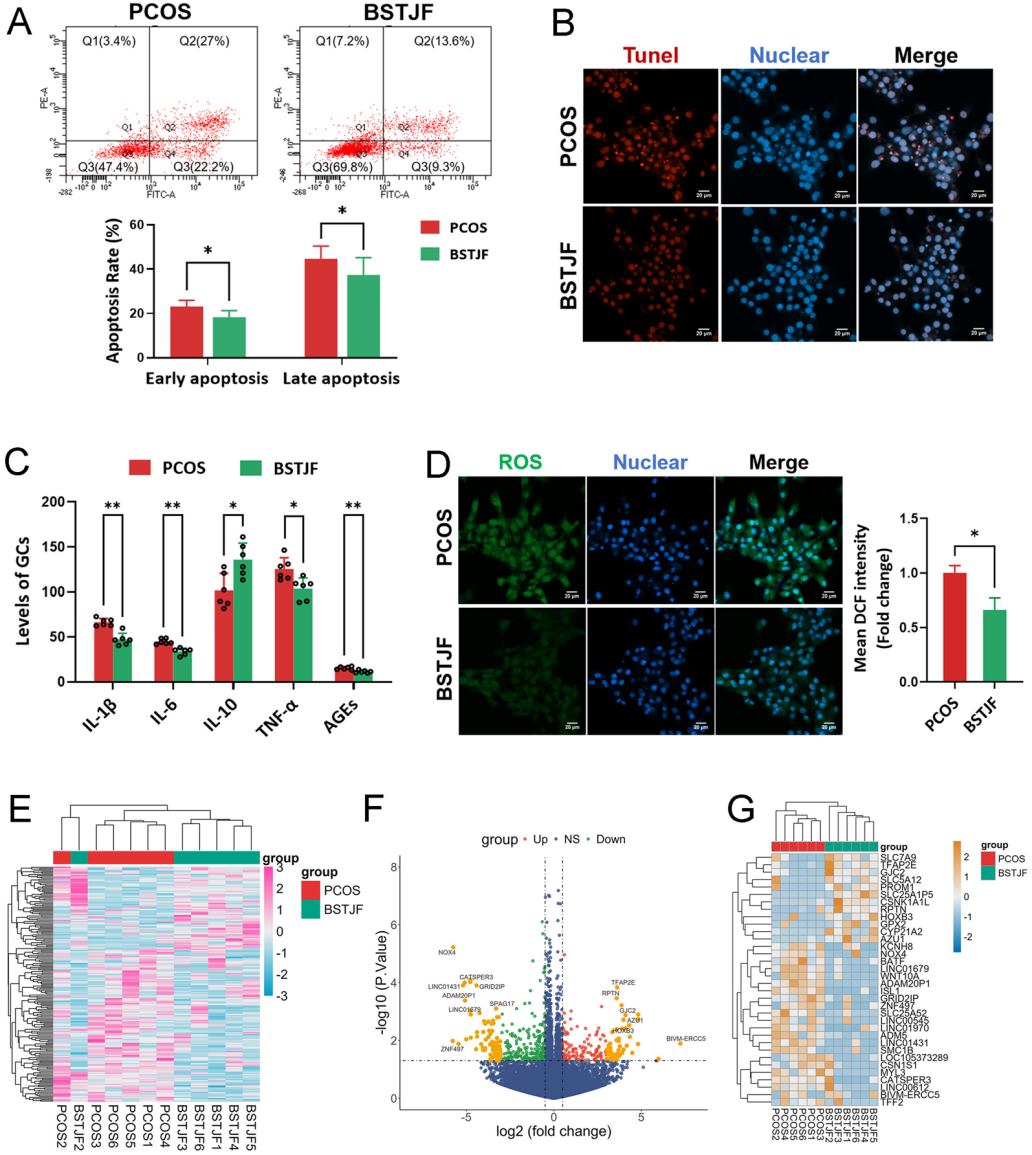
Effects of BSTJF-containing serum on granulosa cells (GCs) from patients with PCOS in vitro. **A** Apoptosis analysis by flow cytometry, with quantification of early and late apoptotic cells in the PCOS and BSTJF groups. Each experiment was independently repeated six times. *P < 0.05. **B** TUNEL fluorescence staining in GCs. **C** Concentrations of interleukin-1 beta (IL-1β), interleukin-6 (IL-6), interleukin-10 (IL-10), tumor necrosis factor-alpha (TNF-α), and advanced glycation end-products (AGEs) in the PCOS and BSTJF groups. *P < 0.05, **P < 0.01. **D** Intracellular ROS levels in GCs, with fluorescence intensity quantification comparing the PCOS and BSTJF groups. Each experiment was independently repeated three times. *P < 0.05. **E** Heatmap showing hierarchical clustering of differentially expressed genes (DEGs) between the PCOS and BSTJF groups. **F** Volcano plot illustrating DEGs between the two groups. **G** Hierarchical clustering analysis of 34 DEGs with |log₂FC|> 3.5

We initially evaluated the effects of BSTJF on GCs isolated from patients with PCOS under in vitro conditions. Human GCs exhibited irregular morphologies and formed clusters after culture, with FSHR, a specifically expressed in GCs and serving as a molecular marker for GC identification, further confirmed by fluorescence staining (Supplemental Fig. 1A). Flow cytometric analysis indicated that BSTJF treatment led to a notable reduction in the percentages of both early and late apoptotic GCs (both *P* < 0.01) (Fig. 2A). The anti-apoptotic effect of BSTJF was further corroborated by TUNEL fluorescence staining, which showed a significant decrease in DNA fragmentation within the GC nuclei (Fig. 2B). Moreover, BSTJF markedly suppressed the production of pro-inflammatory cytokines (IL-1β, IL-6, TNF-α) and AGEs in PCOS-derived GCs, while promoted the the secretion of anti-inflammatory cytokine IL-10 (Fig. 2C). Using the fluorescent probe DCFH-DA, we also observed that BSTJF significantly attenuated intracellular ROS accumulation in PCOS GCs (*P* < 0.05) (Fig. 2D).

**Fig. 3:**
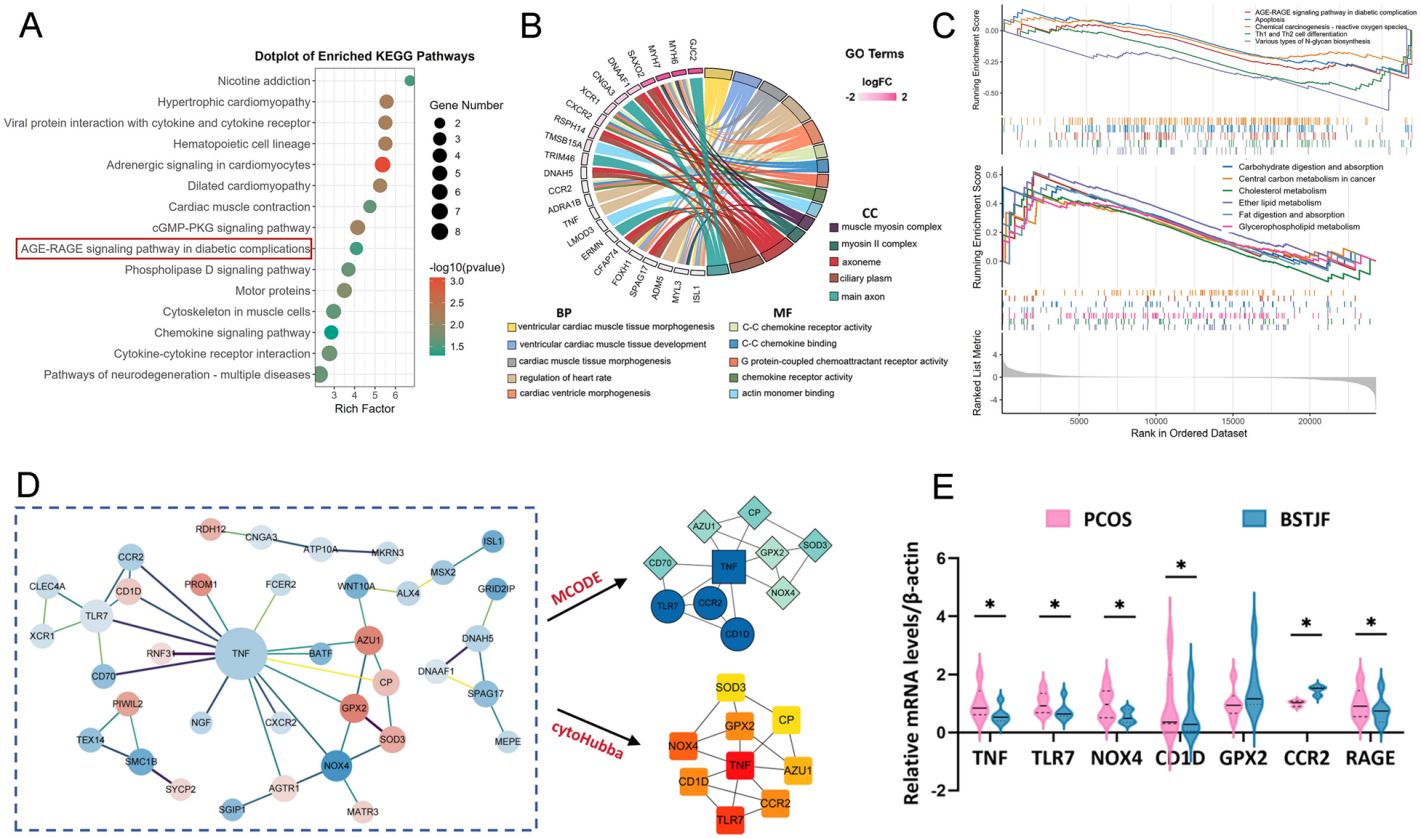
Transcriptomics analysis of granulosa cells (GCs) from PCOS patients treated with BSTJF-containing serum in vitro. **A** Kyoto Encyclopedia of Genes (KEGG) pathway enrichment analysis of DEGs. **B** Chord diagram displaying the top five entries from Gene Ontology (GO) enrichment analysis categories: biological process (BP), molecular function (MF), and cellular component (CC). **C** Gene set enrichment analysis (GSEA) enrichment analysis of key signaling pathways between the transcriptome groups. **D** Protein–protein interaction (PPI) network of DEGs. Red nodes represent upregulated genes, blue nodes represent downregulated genes, with color intensity corresponding to the fold-change. Hub genes were identified using cytoHubba and MCODE. **E** Validation of hub genes (TNF, TLR7, NOX4, CD1D, GPX2 and CCR2) and RAGE expression by RT-qPCR in BSTJF-treated PCOS GCs. *P < 0.05

Transcriptomic analysis identified 195 DEGs in the BSTJF-treated group versus PCOS controls, comprising 74 upregulated and 121 downregulated transcripts. Expression patterns were visualized via hierarchical clustering (Fig. 2E) and volcano plot analysis (Fig. 2F), with 34 genes exhibiting |log2FC|> 3.5 (Fig. 2G). As shown in Fig. 3A, KEGG pathway enrichment revealed significant involvement of DEGs in 14 pathways (*P* < 0.05), prominently including AGE-RAGE signaling in diabetic complications, cytokine-cytokine receptor interactions, and chemokine signaling. GO analysis demonstrated functional stratification of DEGs (Fig. 3B). Integrated functional annotation linked these DEGs to immune-endocrine crosstalk, redox homeostasis, and neurodegenerative pathophysiology, aligning with BSTJF’s therapeutic effects on PCOS-associated metabolic and inflammatory dysregulation.

GSEA of BSTJF-treated PCOS GCs revealed distinct pathway modulation (Fig. 3C). Hallmark gene sets showed upregulation of lipid pathways, including ether lipid metabolism, cholesterol homeostasis, and glycerophospholipid remodeling, alongside nutrient absorption processes (fat and carbohydrate). Conversely, downregulation was observed in pathways linked to catabolic stress including N-glycan biosynthesis, AGE-RAGE signaling, Th1/Th2 differentiation, and oxidative carcinogenesis. Apoptotic signaling was also suppressed. These findings align with BSTJF’s effects in rectifying PCOS-associated metabolic dysregulation, attenuating inflammatory-immune hyperactivation, and restoring redox equilibrium.

A PPI network was constructed from DEGs using the STRING online database and visualized in Cytoscape. Hub genes were identified via cytoHubba (degree centrality) and MCODE (cluster analysis) plugins. As illustrated in Fig. 3D, the hub genes were primarily involved in oxidative stress regulation (NOX4, SOD3, GPX2, CP) and inflammatory response modulation (TNF, TLR7, CCR2, CD1D, AZU1). In the BSTJF-treated group, the oxidative enzyme NOX4 was downregulated while antioxidant enzymes SOD3 and GPX2 were upregulated. Concurrently, pro-inflammatory factors including TNF, TLR7, CCR2, and CD70 exhibited downregulation. The differential expression of these hub genes, as well as that of RAGE, was further validated by RT-qPCR in BSTJF-treated GCs (Fig. 3E). These findings collectively suggest that BSTJF exerts therapeutic effects through antioxidant and anti-inflammatory mechanisms.

### Network pharmacology identifies the AGE-RAGE signaling as a therapeutic pathway of BSTJF in PCOS

Thirteen bioactive components in BSTJF-containing serum were identified by matching precise molecular weights and retention times to authenticated reference standards (Table 1). These included catalpol, danshensu, geniposidic acid, salidroside, p-coumaric acid, psoralenoside, sinapinic acid, isopsoralenoside, astragalin, luteolin, erythrinin C, corylidin and psoralenol/methyl tanshinonate.

**Table 1:** The active components in BSTJF-containing serum detected by UPLC-Q-TOF-MS

NO	t_R_ (min)	Identification	Formula	Detected *m/z*	ppm
1	5.449	Catalpol	C_15_H_22_O_10_	361.1140[M–H]^−^	− 5.0
2	10.835	Danshensu	C_9_H_10_O_5_	197.04[M–H]^−^	1.2
3	14.813	Geniposidic acid	C_16_H_22_O_10_	373.1140[M–H]^−^	− 2.2
4	18.251	Salidroside	C_14_H_20_O_7_	299.1136[M–H]^−^	− 2.1
5	34.420	p-Coumaric Acid	C_9_H_8_O_3_	163.0401[M–H]^−^	6.9
6	38.672	Psoralenoside	C_17_H_18_O_9_	365.0878[M–H]^−^	− 3.9
7	41.526	Sinapinic acid	C_11_H_12_O_5_	223.0612[M–H]^−^	0.5
8	41.850	Isopsoralenoside	C_17_H_18_O_9_	365.0878[M–H]^−^	− 4.1
9	56.303	Astragalin	C_21_H_20_O_11_	447.0933[M–H]^−^	− 6.5
10	57.675	Luteolin	C_15_H_10_O_6_	285.0405[M–H]^−^	4.0
11	63.263	Erythrinin C	C_20_H_18_O_6_	353.1031[M–H]^−^	− 7.0
12	65.844	Corylidin	C_20_H_16_O_7_	367.0823[M–H]^−^	− 9.6
13	68.842	Psoralenol/Methyl tanshinonate	C_20_H_18_O_5_	337.1081[M–H]^−^	− 4.0

**Fig. 4:**
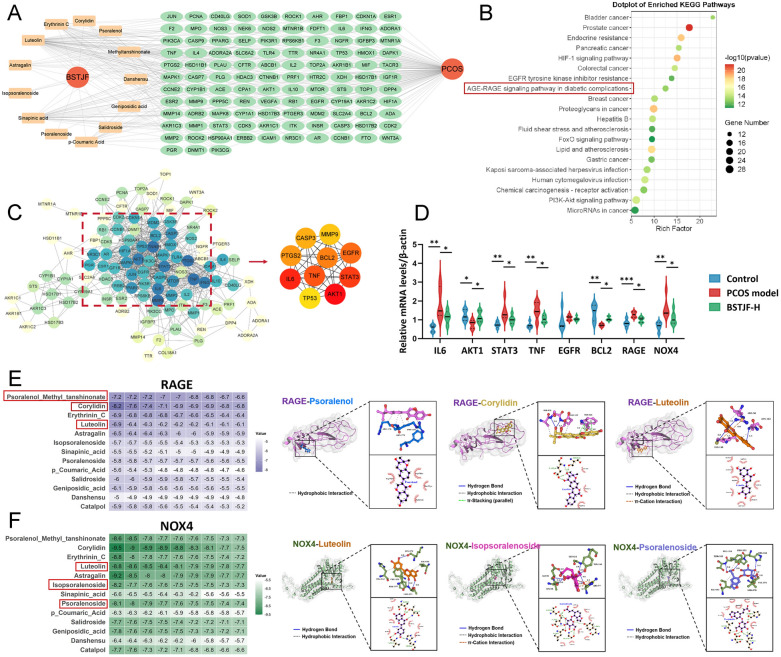
Network pharmacology analysis of BSTJF for the treatment of PCOS. **A** Compound-disease-target network of BSTJF for PCOS treatment. **B** Kyoto Encyclopedia of Genes (KEGG) pathway enrichment analysis of potential therapeutic targets. **C** Protein–protein interaction (PPI) network of potential therapeutic targets. Node color intensity represents the node degree distribution; hub targets were identified using MCODE. **D** Validation of hub genes (IL-6, AKT1, STAT3, TNF, EGFR and BCL2) and potential targets (RAGE and NOX4) by RT-qPCR in a PCOS mouse model after BSTJF treatment. *P < 0.05, **P < 0.01, ***P < 0.001. **E** Molecular docking of bioactive compounds from BSTJF-containing serum with RAGE. The heatmap displays docking scores for 13 active compounds; representative binding conformations are shown for the most stable complexes (psoralenol/methyl tanshinonate, corylidin, luteolin). **F** Molecular docking of bioactive compounds with NOX4. The heatmap shows docking scores for 13 compounds; representative docking poses are displayed for stable binding pairs (luteolin, isopsoralenoside, psoralenoside)

Network pharmacology was employed to predict the multi-target mechanisms through which BSTJF ameliorates PCOS. A total of 345 potential targets of BSTJF bioactive compounds (Supplemental Fig. 2A) were retrieved from the compound-target databases. PCOS-related targets (1568 genes) were collated from the disease databases. Cross-referencing these datasets identified 113 overlapping therapeutic targets (Supplemental Fig. 2B), visualized as a compound-disease-target network (Fig. 4A). Functional enrichment analysis of shared targets highlighted BSTJF’s multi-pathway regulatory capacity, with KEGG pathway profiling revealing significant enrichment in the AGE-RAGE, HIF-1, FoxO, and PI3K-Akt signaling pathways (Fig. 4B). GO analysis revealed predominant involvement in testosterone dehydrogenase [NAD(P)] activity, steroid dehydrogenase activity, protein phosphatase binding and response to oxidative stress (Supplemental Fig. 2C). A PPI network of targets was constructed and further analysis with CytoHubba identified six hub targets including IL-6, AKT1, STAT3, TNF, EGFR and BCL2 (Fig. 4C), which converge on AGE-RAGE signaling to regulate inflammation and apoptosis. The differential expression of these hub genes, as well as that of RAGE and NOX4, was further validated by RT-qPCR in a PCOS mouse model treated with BSTJF (Fig. 4D). These findings suggest that BSTJF ameliorates PCOS pathophysiology with the AGE-RAGE signaling serving as an important regulatory pathway.

Molecular docking simulations were conducted to evaluate interactions between 13 bioactive BSTJF serum components and core targets in the AGE-RAGE pathway. As the main candidate target proteins, RAGE and NOX4 were subjected to molecular docking with compounds. Binding affinities (kcal/mol) were quantified for each compound-target pair, with heatmaps visualizing docking scores across RAGE (Fig. 4E) and NOX4 (Fig. 4F) conformational ensembles. Strong binding (< − 7.0 kcal/mol) was observed for Corylidin (RAGE: − 8.2; NOX4: − 9.5), Psoralenol/Methyl_tanshinonate (RAGE: − 7.2; NOX4: − 8.6), Astragalin (NOX4: − 9.2), and Erythrinin C/Luteolin (NOX4: − 8.8 each). Notably, previously reported BSTJF core components—isopsoralenoside, psoralenoside, and salidroside [16, 17]—exhibited moderate-to-high affinity for both targets (− 5.8 to − 7.1 kcal/mol). These data support RAGE and NOX4 as therapeutic targets of BSTJF in PCOS.

### BSTJF improves estrous cycle, glucose tolerance and hormonal profile in PCOS mice

**Fig. 5:**
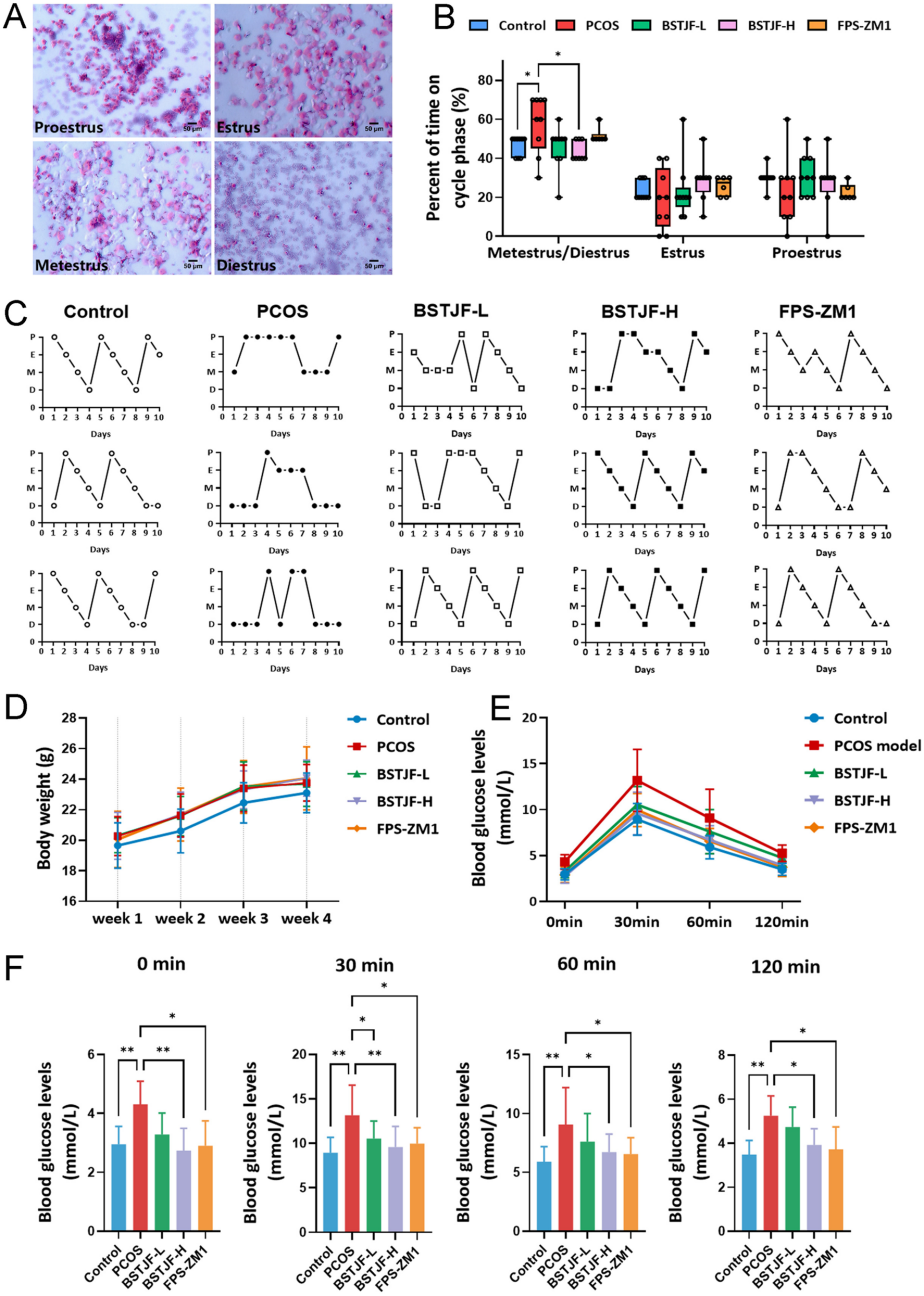
Estrous cycle, body weight and glucose tolerance in PCOS mouse model intervened by BSTJF. **A** Representative photomicrographs of the four estrus stages of the estrous cycle of a female mouse; Bars = 50 μm. **B** The percent of mice on different stages of estrous cycle in each group (%). *P < 0.05. **C** Representative images of estrous cycle over 10 days from each group. P, proestrus; E, estrus; M, metestrus; D, diestrus. **D** Weekly body weight monitoring over 4 weeks in from each group. **E** Intraperitoneal glucose tolerance testing (IPGTT) in mice from each group. **F** Comparison of blood glucose levels at each timepoint (0, 30, 60, 120 min) between the groups. *P < 0.05, **P < 0.01

A DHEA-induced murine PCOS model was employed to evaluate BSTJF’s therapeutic efficacy. Given that RAGE is an important target of BSTJF and existing literature has demonstrated that RAGE inhibitors can ameliorate PCOS [35] and regulate the phosphorylation of p65 NF-κB and p38 MAPK signaling [38], we employed the RAGE inhibitor FPS-ZM1 as a positive control in the animal experiments. Vaginal cytology (Fig. 5A) revealed prolonged metestrus-diestrus phases in PCOS mice (60% vs. 50% in controls), indicative of cycle disruption. Both low/high-dose BSTJF and FPS-ZM1 restored regular 4–5 day estrous cyclicity, with high-dose BSTJF achieving near-normal phase distribution comparable to controls (Fig. 5B, C).

Weekly body weight monitoring over 4 weeks revealed modest weight gain in DHEA-induced PCOS mice versus controls, though intergroup differences were not statistically significant (Fig. 5D). IPGTT demonstrated marked glucose intolerance in PCOS mice, with elevated blood glucose at all timepoints (0 min: 4.3 ± 0.8 vs. 3.0 ± 0.6 mmol/L; 120 min: 5.3 ± 0.9 vs. 3.5 ± 0.6 mmol/L; *P* < 0.01 vs. control). High-dose BSTJF and FPS-ZM1 interventions normalized glycemia at all timepoints (all *P* < 0.05 vs. PCOS), while low-dose BSTJF reduced 30-min post-load glucose (10.5 ± 2.0 vs. 13.2 ± 3.4 mmol/L; *P* < 0.05 vs. PCOS) (Fig. 5E, F).

**Fig. 6:**
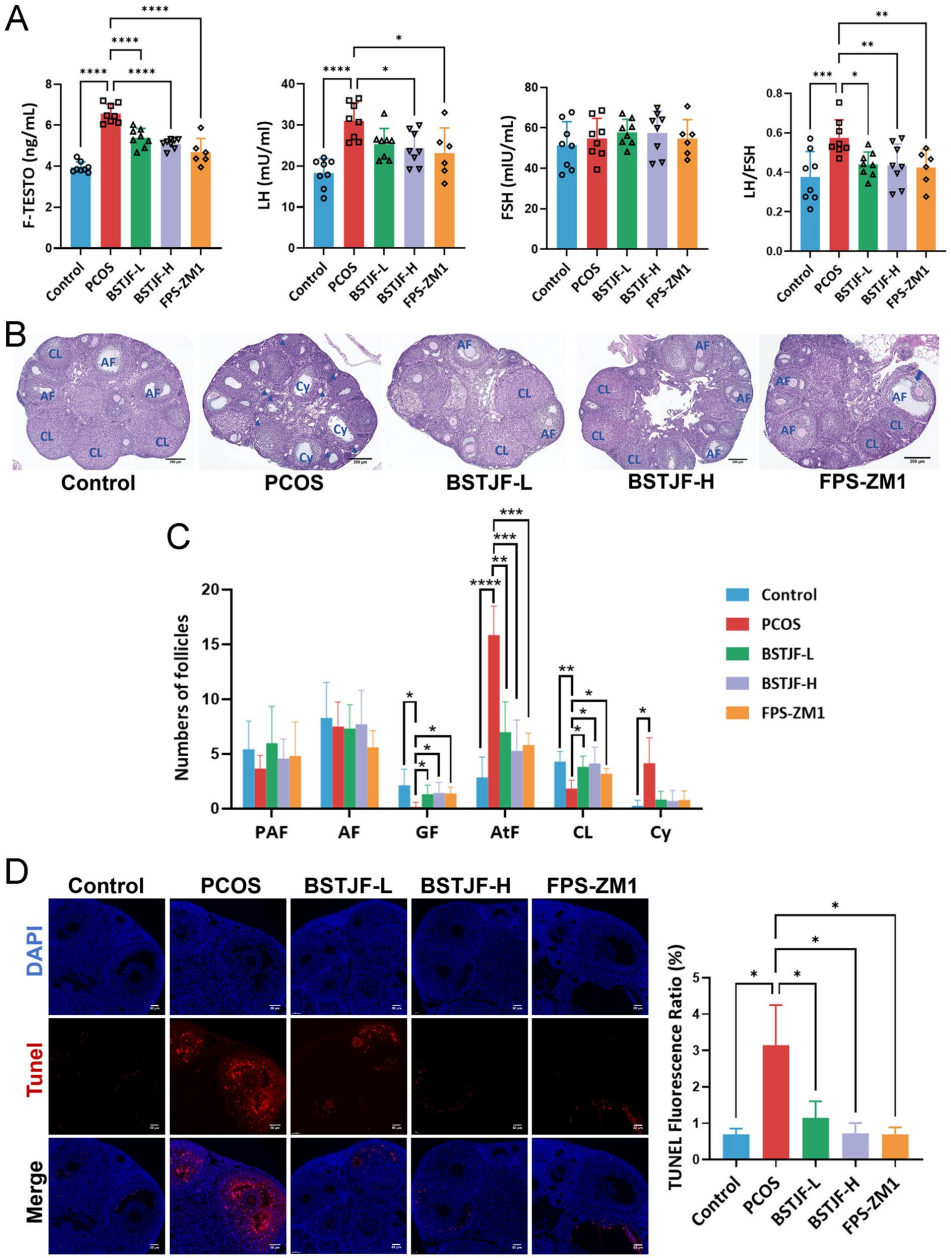
Hormonal profile, ovarian folliculogenesis and granulosa cell apoptosis in PCOS mouse model intervened by BSTJF. **A** Serum levels of free testosterone, luteinizing hormone (LH), follicle-stimulating hormone (FSH) and LH/FSH among the groups. *P < 0.05, **P < 0.01, ***P < 0.001, ****P < 0.0001. **B** Representative micrographs of ovarian sections. AF, antral follicle; CL, corpus luteum; Cy, ovarian cysts; triangles denote atretic follicles, and an arrow indicates a Graafian follicle. Bars = 200 μm. **C** Counts of follicles at different developmental stages. PAF, pre-antral follicle; PF, primordial follicle; AF, antral follicle; GF, Graafian follicle; AtF, atretic follicle; CL, corpus luteum; CF, cystic follicle. Data were all presented as mean ± SD, *P < 0.05, **P < 0.01, ***P < 0.001, ****P < 0.0001. **D** TUNEL staining-positive granulosa cells and the comparison of TUNEL fluorescence ratio (%) among the groups. Data were all presented as mean ± SD, *P < 0.05, **P < 0.01

PCOS mice exhibited hyperandrogenemia (free testosterone: 6.6 ± 0.5 vs. 3.9 ± 0.3 ng/mL) and LH elevation (31.0 ± 4.5 vs. 18.3 ± 3.5 mU/mL) versus controls (both *P* < 0.01). Both high-dose BSTJF and FPS-ZM1 restored testosterone (5.1 ± 0.2 and 4.7 ± 0.7 ng/mL) and LH (24.3 ± 4.2 and 23.2 ± 6.1 mU/mL) levels (*P* < 0.05 vs. PCOS) (Fig. 6A). Although FSH remained unchanged across groups, the elevated LH/FSH ratio in PCOS mice (0.6 ± 0.1 vs. 0.3 ± 0.1) was attenuated by low-dose BSTJF (0.4 ± 0.1, *P* < 0.05), high-dose BSTJF (0.4 ± 0.1, *P* < 0.01), and FPS-ZM1 (0.4 ± 0.1, *P* < 0.01), indicating improved ovarian reserve.

### BSTJF restores ovarian function and mitigates granulosa cell apoptosis in PCOS mice

Histopathological evaluation of ovarian tissues was conducted to validate successful PCOS induction and assess BSTJF’s therapeutic impact on ovarian folliculogenesis. Representative micrographs of ovarian sections (Fig. 6B) demonstrated well-developed antral follicles and corpora lutea in control mice. In contrast, DHEA-induced PCOS ovaries exhibited pathological hallmarks including frequent atretic follicles, cystic follicles, diminished Graafian follicles and reduced corpora lutea, confirming folliculogenesis impairment. Quantitative follicular staging (Fig. 5C) revealed comparable pre-antral and antral follicle counts across groups. However, PCOS mice showed a marked depletion of Graafian follicles (*P* < 0.01) and increased atretic follicles (*P* < 0.01). Low-and high-dose BSTJF, alongside FPS-ZM1, significantly restored Graafian follicle numbers (all *P* < 0.05 vs. PCOS) while reducing atretic follicles (all *P* < 0.01). Corpora lutea counts, severely diminished in PCOS mice (*P* < 0.01), were partially rescued by all treatments (all *P* < 0.05 vs. PCOS). As shown in Supplemental Fig. 1B, ovarian GCs in mice were also identified using FSHR immunofluorescence staining.

The functional integrity of ovarian GCs is critical for follicular maturation, with GC apoptosis being implicated in the pathogenesis of atretic follicle formation and ovulatory dysfunction in PCOS [39]. Consistent with this mechanism, TUNEL staining demonstrated a significant elevation in TUNEL-positive GCs within PCOS ovaries compared to controls (*P* < 0.01), confirming DHEA-induced apoptotic activation (Fig. 6D). Notably, low-dose BSTJF, high-dose BSTJF, and FPS-ZM1 treatment significantly decreased the proportion of apoptotic GCs (*P* < 0.05, *P* < 0.01, and *P* < 0.01, respectively), providing evidence that both BSTJF and FPS-ZM1 mitigate GC apoptosis in PCOS mice.

### BSTJF attenuates inflammation and oxidative damage in PCOS mice

**Fig. 7:**
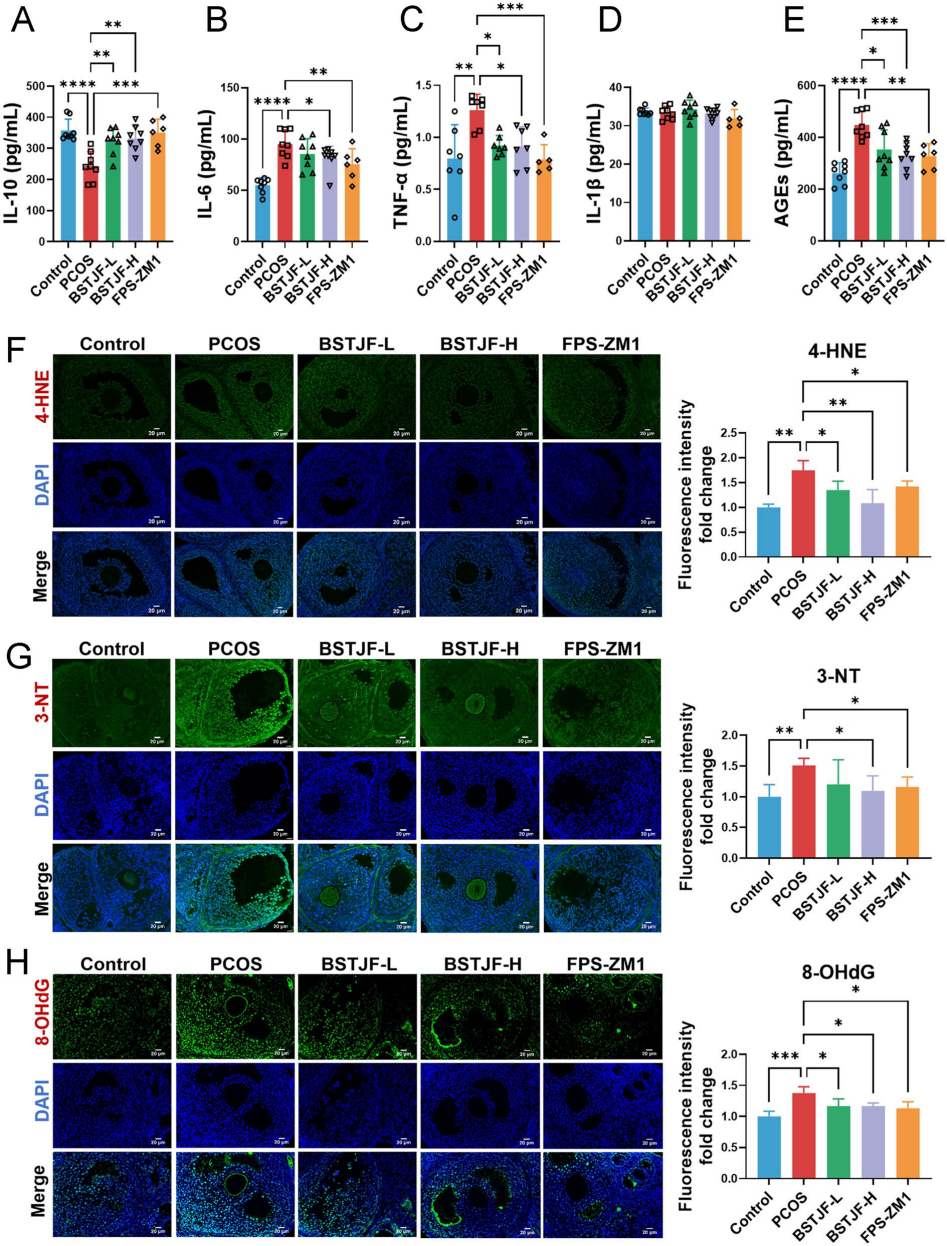
Effects of BSTJF on inflammation and oxidative stress in PCOS mice. **A**–**E** Serum concentrations of interleukin-10 (IL-10), interleukin-6 (IL-6), tumor necrosis factor-alpha (TNF-α), interleukin-1 beta (IL-1β) and advanced glycation end-products (AGEs) among the groups. *P < 0.05, **P < 0.01, ***P < 0.001, ****P < 0.0001. **F**–**G** Immunofluorescence of the biomarker of lipid peroxidation, protein oxidation and oxidative DNA damage by detecting 4-Hydroxynonenal (4-HNE), 3-Nitrotyrosine (3-NT) and 8-Hydroxy-2′-deoxyguanosine (8-OHdG), respectively, and the comparison of fluorescence quantification among the groups. Data were all presented as mean ± SD, *P < 0.05, **P < 0.01, ***P < 0.001

Evidence have shown that oxidative stress and inflammation are closely related to the molecular pathogenesis of PCOS, and our previous study has demonstrated abnormal serum levels of GSH, MDA and SOD in PCOS animal model and BSTJF could reverse these biomarkers of oxidative stress [16]. Here we further examined inflammatory factors and it has been showed PCOS mice had markedly increased serum levels of IL-6 and TNF-α, and decreased serum level of IL-10, which were significantly reversed in BSTJF-H and FPS-ZM1 groups compared with PCOS group (all *P* < 0.05) (Fig. 7A-C). The serum levels of IL-10 and TNF-α could also be significantly reversed by intervention of low-dose BSTJF (*P* < 0.01 and *P* < 0.05), while level of IL-1β was similar among the groups (Fig. 7D). Notably, serum AGEs accumulation was elevated in PCOS mice but significantly attenuated across all BSTJF-treated and FPS-ZM1 groups compared with PCOS group (all *P* < 0.05) (Fig. 7E). These results indicate that BSTJF may restore the ovarian microenvironment in PCOS by mitigating inflammation and reducing AGEs accumulation.

To investigate the effect of BSTJF on oxidative stress in ovarian GCs, we measured the biomarker of lipid peroxidation, protein oxidation and oxidative DNA damage by detecting 4-HNE, 3-NT and 8-OHdG, respectively. Representative images of immunostaining with each oxidative damage marker in GCs are shown in Fig. 7F–H. The levels of 4-HNE, 3-NT and 8-OHdG were dramatically increased in GCs of PCOS mice (all *P* < 0.01), while significantly decreased in a dose-dependent manner after BSTJF treatment (all *P* < 0.05), and FPS-ZM1 also significantly reversed the levels of 4-HNE, 3-NT and 8-OHdG (all *P* < 0.05). Our findings support the hypothesis that accumulating oxidative damage may underlie GC apoptosis and ovulation disorder in PCOS mice, and BSTJF and FPS-ZM1 treatment can modify the excessive oxidative stress state.

### BSTJF ameliorates metabolic disturbances via regulating glucolipid metabolic profiling in PCOS mice

**Fig. 8:**
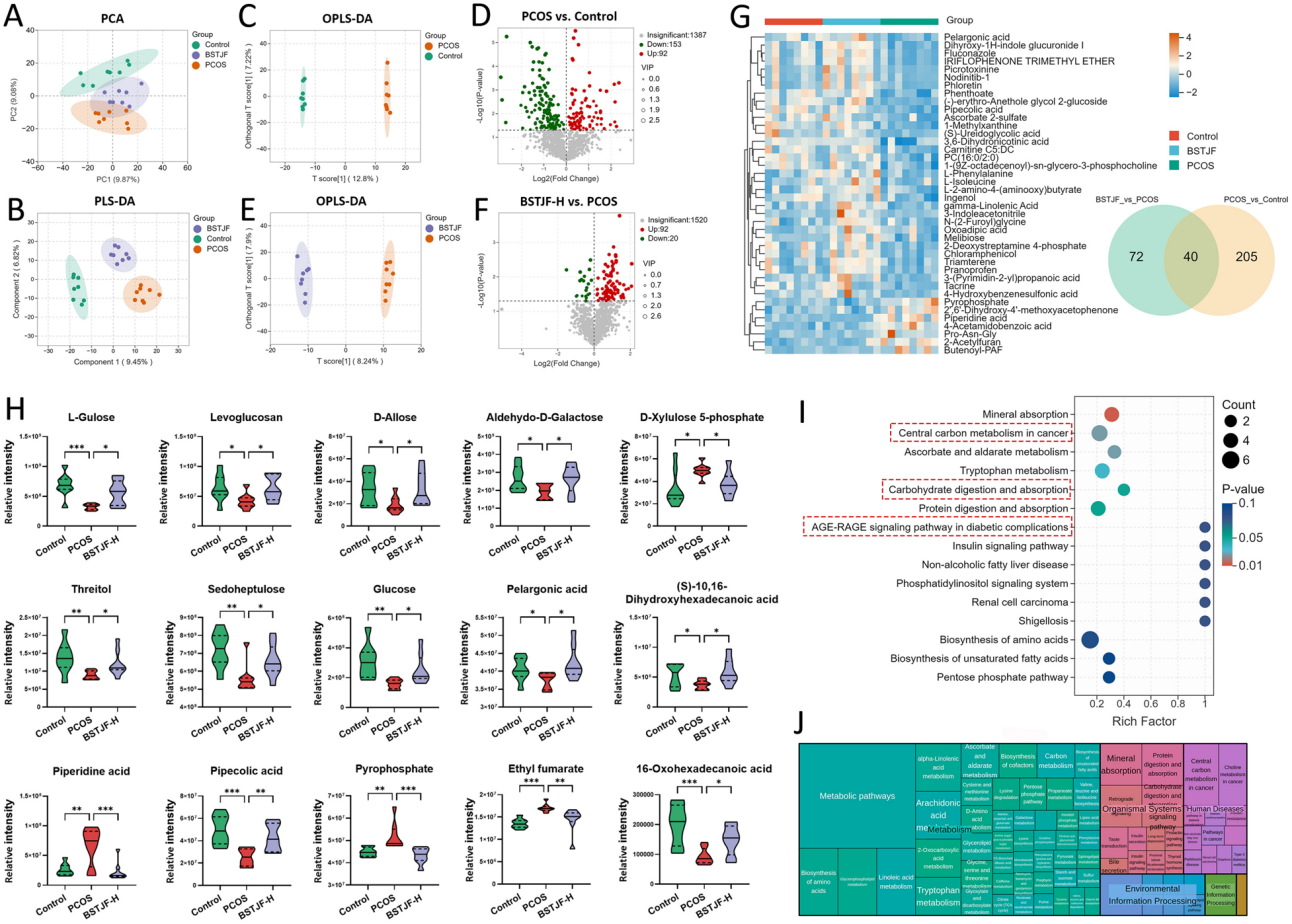
Effects of BSTJF on metabolomic profiling in PCOS mice characterized by untargeted serum metabolomics. **A** Principal component analysis (PCA) score plot of control, PCOS, and BSTJF high-dose treatment groups; **B** Partial least squares-discriminant analysis (PLS-DA) score plot; **C** Orthogonal PLS-DA (OPLS-DA) pairwise comparison between PCOS vs. control groups; **D** Volcano plot of significant differential metabolites between PCOS vs. control groups; **E** OPLS-DA pairwise comparison between BSTJF-H vs. PCOS groups; **F** Volcano plot of significant differential metabolites between BSTJF-H vs. PCOS groups; **G** Core metabolites modulated by BSTJF were identified through Venn diagram intersection and visualized via clustering heatmap; **H** Violin plots of 15 core metabolites exhibiting near-normalization trends post-treatment of BSTJF; Data were all presented as mean ± SD, *P < 0.05, **P < 0.01, ***P < 0.001. **I** Kyoto Encyclopedia of Genes (KEGG) pathway enrichment analysis of core metabolites; Pathways with enrichment significance below *P* < 0.1 are shown. **J** Treemap visualization of KEGG enrichment results. Colors represent major categories, rectangles indicate pathways, and size corresponds to gene count

To systematically investigate the therapeutic mechanisms of BSTJF, we conducted untargeted serum metabolomics to characterize metabolic profiles in control, PCOS, and high-dose BSTJF treatment groups. Principal component analysis (PCA) revealed distinct clustering patterns (Fig. 8A), indicating PCOS-associated metabolic reprogramming and its partial reversal by BSTJF intervention. Supervised partial least squares-discriminant analysis (PLS-DA) further validated robust metabolic separations across groups (R^2^Y = 0.856, Q^2^ = 0.412; Fig. 8B), confirming the reliability of metabolic signature differentiation. Orthogonal PLS-DA (OPLS-DA) pairwise comparisons between PCOS vs. control and BSTJF-H vs. PCOS groups exhibited complete metabolic profile segregation (Fig. 8C, E), with ions meeting VIP > 1.0 and *P* < 0.05 thresholds classified as significant differential metabolites. Comparative analysis revealed 245 dysregulated metabolites in PCOS vs. controls (Fig. 8D) and 112 BSTJF-responsive metabolites (Fig. 8F). Intersection analysis through Venn diagram revealed 40 core metabolites critically modulated by BSTJF (Fig. 8G; Table 2), 15 of which exhibited near-normalization trends post-treatment (Fig. 8H). The statistical analysis for the reversal of these 15 metabolites is provided in Supplementary Table 2. These metabolites could be predominantly clustered into carbohydrate metabolism regulators (L-gulose, sedoheptulose, levoglucosan, D-allose), lipid homeostasis mediators (pelargonic acid, S-10,16-dihydroxyhexadecanoic acid, 16-oxohexadecanoic acid), and bioactive organic acids (pipecolic acid, pyrophosphate, and fumarate derivatives). KEGG pathway analysis mapped the core metabolites to key metabolic axes implicated in PCOS and modulated by BSTJF intervention, including core energy metabolism (carbon metabolism, carbohydrate digestion/absorption), insulin sensitization networks (insulin signaling, AGE-RAGE axis), and antioxidant defense (ascorbate/aldarate metabolism) (Fig. 8I; Supplementary Table 3). The functional landscape, outlined by a treemap visualization (Fig. 8J), positioned pathways central to metabolism. These findings suggest BSTJF's therapeutic potential in PCOS-related glucolipid dysregulation.

**Table 2:** The differential metabolites and their trends after BSTJF administration

NO	Metabolite	Formula	RT (min)	Measured *m/z*	Variation trend	
					PCOS vs. Control	BSTJF vs. PCOS
1	Pipecolic acid	C_6_H_11_NO_2_	1.0755	130.0866	↓***	↑^###^
2	L-Gulose	C_6_H_12_O_6_	0.7824	203.0527	↓**	↑^#^
3	1-(9Z-octadecenoyl)-sn-glycero-3-phosphocholine	C_26_H_52_NO_7_P	7.1773	544.3372	↓**	↑^#^
4	3-Formylsalicylic acid	C_8_H_6_O_4_	1.3952	149.0239	↓**	↑^#^
5	1,5-Anhydro-D-fructose	C_6_H_10_O_5_	0.7824	185.0423	↓**	↑^##^
6	1-Stearoyl-2-arachidonoylglycerol	C_41_H_72_O_5_	8.9166	627.5331	↓**	↑^#^
7	(-)-erythro-Anethole glycol 2-glucoside	C_16_H_24_O_8_	0.7948	383.1154	↓***	↑^#^
8	7alpha,12alpha,26-Trihydroxycholest-4-en-3-one	C_27_H_44_O_4_	8.9291	496.3385	↓*	↑^#^
9	PC(16:0/2:0)	C_26_H_52_NO_8_P	7.1482	520.3378	↓**	↑^#^
10	Carnitine C5:DC	C_12_H_21_NO_6_	4.9642	315.1337	↓*	↑^#^
11	Ingenol	C_20_H_28_O_5_	4.8111	347.1892	↓**	↑^#^
12	Prostaglandin K1	C_20_H_32_O_5_	4.8874	351.2205	↓**	↑^#^
13	Pelargonic acid	C_9_H_18_O_2_	5.4617	157.1234	↓*	↑^#^
14	Levoglucosan	C_6_H_10_O_5_	0.7664	161.0455	↓*	↑^#^
15	D-Allose	C_6_H_12_O_6_	1.2021	161.0455	↓*	↑^#^
16	Threitol	C_4_H_10_O_4_	0.7541	143.0347	↓*	↑^#^
17	3-(Pyrimidin-2-yl)propanoic acid	C_7_H_8_N_2_O_2_	2.2215	151.0611	↓*	↑^#^
18	1-Methylxanthine	C_6_H_6_N_4_O_2_	4.4423	225.0774	↓**	↑^##^
19	4-Hydroxybenzenesulfonic acid	C_6_H_6_O_4_S	3.1501	172.974	↓**	↑^#^
20	Aldehydo-D-Galactose	C_6_H_12_O_6_	0.9776	215.0329	↓*	↑^#^
21	(S)−10,16-Dihydroxyhexadecanoic acid	C_16_H_32_O_4_	6.7655	269.2128	↓*	↑^#^
22	Tacrine	C_13_H_14_N_2_	4.455	198.1136	↓*	↑^#^
23	Sedoheptulose	C_7_H_14_O_7_	0.8193	269.0894	↓**	↑^#^
24	Phenthoate	C_12_H_17_O_4_PS_2_	0.7541	354.9956	↓**	↑^##^
25	(S)-Ureidoglycolic acid	C_3_H_6_N_2_O_4_	0.9097	155.0116	↓*	↑^#^
26	16-Oxohexadecanoic acid	C_16_H_30_O_3_	7.2624	269.2132	↓**	↑^#^
27	Glucose	C_6_H_12_O_6_	0.7664	179.0559	↓**	↑^#^
28	3,6-Dihydronicotinic acid	C_6_H_7_NO_2_	9.1189	124.0402	↓*	↑^#^
29	Ascorbate 2-sulfate	C_6_H_8_O_9_S	1.1149	254.9823	↓*	↑^#^
30	Piperidine acid	C_6_H_11_NO_2_	0.7948	130.0866	↑**	↓^##^
31	2-Acetylfuran	C_6_H_6_O_2_	5.2902	111.0394	↑***	↓^#^
32	Ribavirin	C_8_H_12_N_4_O_5_	0.9695	244.0927	↑*	↓^#^
33	2′,6′-Dihydroxy-4′-methoxyacetophenone	C_9_H_10_O_4_	5.3735	183.062	↑*	↓^#^
34	4-Acetamidobenzoic acid	C_9_H_9_NO_3_	4.6455	179.0526	↑**	↓^#^
35	(1R,4S,5S)−6-methoxycyclohexane-1,2,3,4,5-pentol	C_7_H_14_O_6_	0.6698	271.0034	↑***	↓^#^
36	2-O-(6-Phospho-alpha-mannosyl)-D-glycerate	C_9_H_17_O_12_P	0.6698	433.0062	↑***	↓^##^
37	D-Xylulose 5-phosphate	C_5_H_11_O_8_P	1.0891	264.9902	↑*	↓^#^
38	Arsanilic acid	C_6_H_8_AsNO_3_	8.9384	215.9607	↑*	↓^##^
39	Pyrophosphate	H_4_O_7_P_2_	0.7324	176.9285	↑*	↓^##^
40	Ethyl fumarate	C_6_H_8_O_4_	0.6553	143.0207	↑***	↓^#^

Statistical significance was evaluated using an unpaired student’s t-test and *P* < 0.05 was selected as discrimination of significant difference.***P < 0.001, **P < 0.01, and *P < 0.05, when versus Control in serum samples; ^###^ *P* < 0.001, ^##^*P* < 0.01, and ^#^ *P* < 0.05, when versus PCOS in serum samples. ↑indicates up-regulation; ↓indicates down-regulation

### BSTJF suppresses AGEs-RAGE-driven NOX4 activation in PCOS mice

**Fig. 9:**
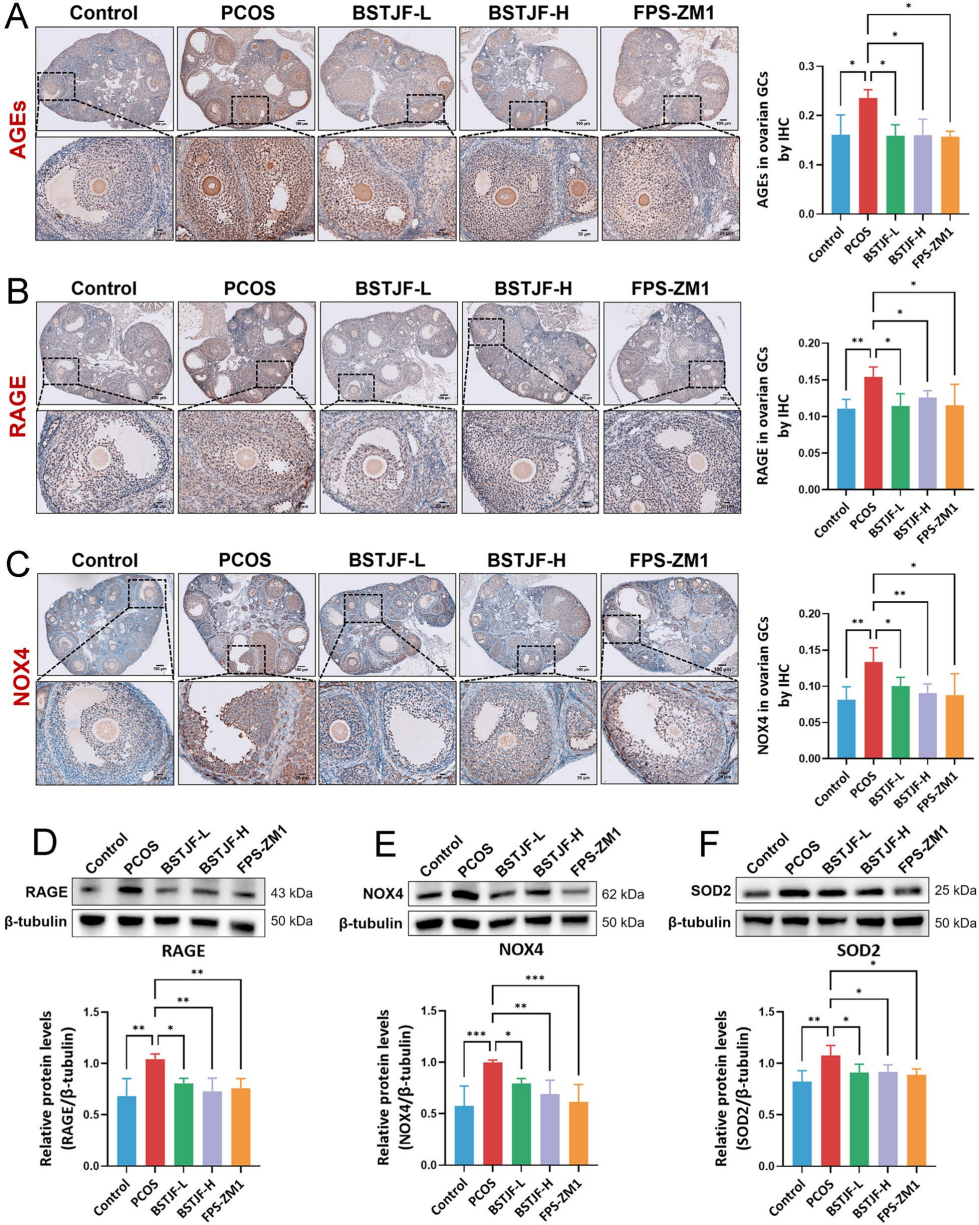
BSTJF suppresses AGEs-RAGE-driven NOX4 activation in PCOS mice. **A**–**C** Immunohistochemical analysis of AGEs accumulation, RAGE and NOX4 expressions in ovarian GCs across the groups. **D**–**F** Western blot analysis of protein expressions of RAGE, NOX4 and SOD2 in ovarian tissues among groups. Data were all presented as mean ± SD, *P < 0.05, **P < 0.01, ***P < 0.001

To confirm whether AGEs plays a role in PCOS pathophysiology via RAGE activation, we quantified the AGEs-RAGE axis in ovarian tissues across the groups. Immunohistochemical analysis revealed significantly elevated AGEs accumulation in ovarian GCs of PCOS mice compared to controls (*P* < 0.05), which was markedly attenuated by both low-, high-dose BSTJF and the RAGE inhibitor FPS-ZM1 (*P* < 0.05; Fig. 9A). Consistent with this, RAGE expression in ovarian GCs was substantially upregulated in PCOS mice (*P* < 0.01) but significantly downregulated following low-, high-dose BSTJF and FPS-ZM1 interventions (*P* < 0.05; Fig. 9B). Western blot analysis of ovarian tissues further confirmed the RAGE overexpression in PCOS and its suppression by treatments (Fig. 9D). These findings indicate that BSTJF mitigates AGEs overproduction and subsequent AGEs-RAGE axis hyperactivation in PCOS GCs.

We next examined whether BSTJF affects ovarian NOX4 which is known to be activated by AGEs-RAGE signaling to activate intracellular oxidative stress. Immunohistochemistry (Fig. 9C) and western blotting (Fig. 9E) demonstrated pronounced NOX4 overexpression in PCOS ovarian tissues (*P* < 0.01), particularly within GCs, compared to controls. Both low-, high-dose BSTJF and FPS-ZM1 treatments significantly suppressed NOX4 levels (*P* < 0.05). Moreover, superoxide dismutase 2 (SOD2), a ROS-activated mitochondrial antioxidant enzyme, was significantly upregulated in the ovaries of PCOS mice, whereas low-, high-dose BSTJF and FPS-ZM1 treatments markedly suppressed SOD2 overexpression compared to PCOS mice (all *P* < 0.05) (Fig. 9F). Collectively, these data suggest that BSTJF ameliorates oxidative stress in PCOS ovaries, at least partially through inhibiting AGEs-RAGE-driven NOX4 activation.

### BSTJF inhibits p38 MAPK activation and NF-κB nuclear translocation in PCOS mice

**Fig. 10:**
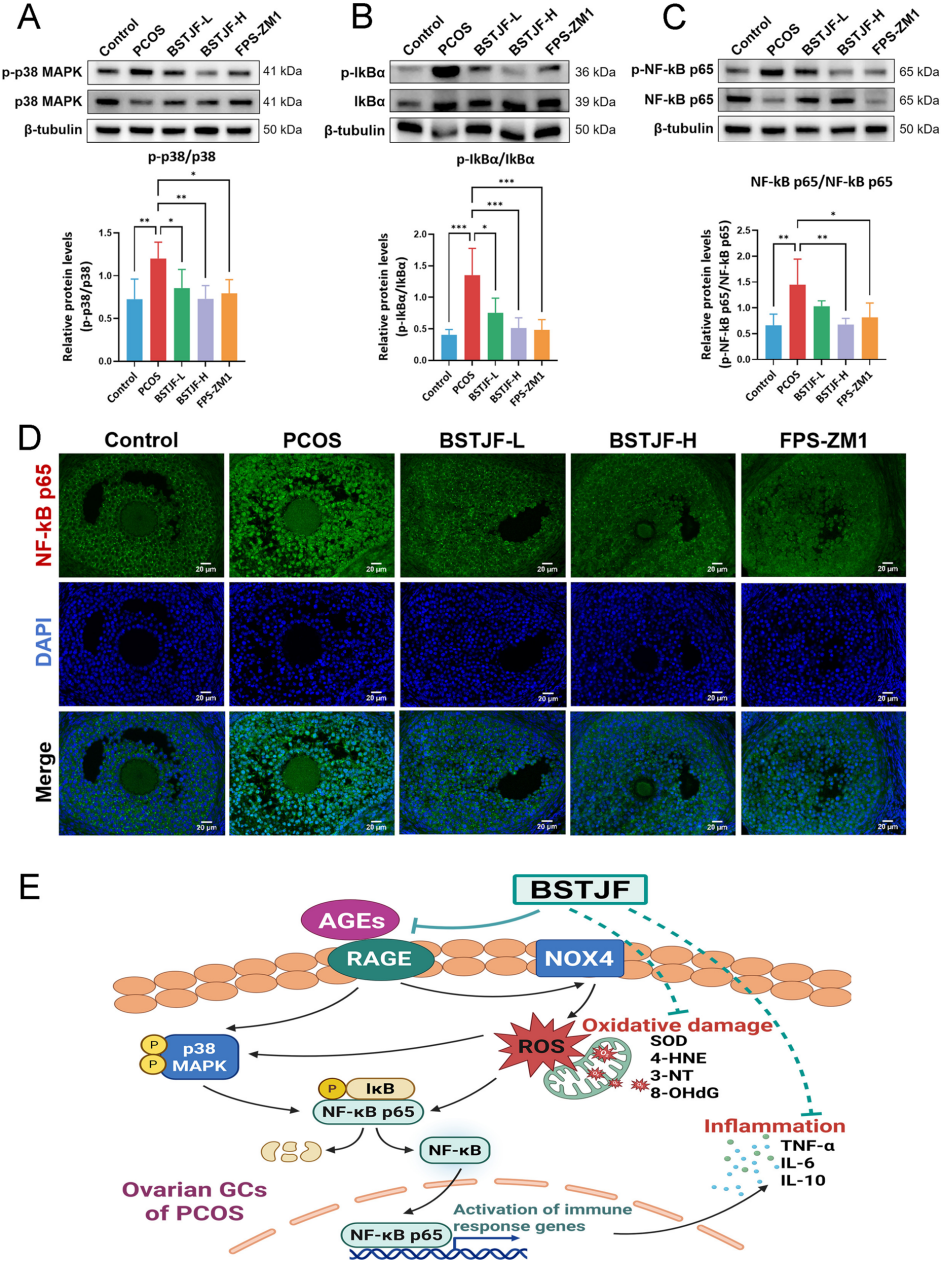
BSTJF inhibits p38 MAPK activation and NF-κB nuclear translocation in PCOS mice. **A**–**C** Western blot analysis of phosphorylated p38 MAPK/p38 MAPK, phosphorylated IκBα/IκBα, and phosphorylated NF-κB p65/NF-κB p65 in ovarian tissues across the groups. Data were all presented as mean ± SD, *P < 0.05, **P < 0.01, ***P < 0.001. **D** Immunofluorescence analysis of nuclear translocation of NF-κB p65 in ovarian GCs. **E** Schematic diagram of the mechanisms by which BSTJF ameliorates oxidative-inflammatory stress in PCOS by regulating the AGEs-RAGE/NOX4/NF-κB pathway. Created in https://BioRender.com

Activation of the AGEs-RAGE axis drives oxidative stress through ROS accumulation while concurrently dysregulating p38 MAPK signaling and activating NF-κB p65, which play crucial roles in the production of multiple pro-inflammatory cytokines. To elucidate BSTJF’s anti-inflammatory mechanisms in PCOS, we assessed its impact on these signaling nodes. Western blot analysis demonstrated significant upregulation of phosphorylated p38 MAPK, IκBα, and NF-κB p65 in PCOS ovarian tissues versus controls (*P* < 0.05; Fig. 10A–C). Both high-dose BSTJF and the RAGE inhibitor FPS-ZM1 effectively normalized these phosphorylation events, while low-dose BSTJF significantly reduced phospho-p38 MAPK and phospho-IκBα levels, with a modest effect on phospho-NF-κB p65. Complementary immunofluorescence staining revealed enhanced nuclear translocation of NF-κB p65 in PCOS ovarian GCs (Fig. 10D), indicative of transcriptional activation of inflammatory mediators. By contrast, low-, high-dose BSTJF and FPS-ZM1 groups exhibited predominant cytoplasmic retention of NF-κB p65 in GCs, effectively blocking its nuclear pro-inflammatory activity. These coordinated findings establish that BSTJF disrupts AGEs-RAGE downstream effectors—p38 MAPK and NF-κB pathways, thereby mitigating inflammatory responses in PCOS.

## Discussion

In the present study, preliminary in vitro experiments indicated that BSTJF reduced apoptosis and ROS generation in GCs from PCOS ovaries. Transcriptomic profiling further revealed significant enrichment of differentially expressed genes involved in the AGEs-RAGE signaling pathway, many of which are associated with oxidative stress and inflammatory responses. Integrated network pharmacology and molecular docking analyses predicted strong binding interactions between bioactive components of BSTJF and the target proteins RAGE and NOX4. Subsequent in vivo validation demonstrated that BSTJF ameliorates GC dysfunction in PCOS likely through targeting the AGEs-RAGE axis to suppress NOX4 activation, leading to inhibition of p38 MAPK phosphorylation and NF-κB nuclear translocation, ultimately alleviating ovarian oxidative and inflammatory stress (Fig. 10E).

Within the ovarian microenvironment, disruptions in inflammatory balance and redox homeostasis promote follicular atresia by inducing GC apoptosis, particularly under PCOS conditions [40]. Emerging therapeutic strategies targeting these microenvironmental perturbations may thus mitigate GC dysfunction and PCOS phenotypes. Recent bioinformatics studies have demonstrated the value of multi-omics analysis in chronic diseases [41] and the efficacy of network pharmacology in identifying drug-gene interactions [42], which collectively supports our integrated methodological strategy. In this study, transcriptomic profiling identified BSTJF-modulated hub genes regulating inflammatory mediators (TNF, TLR7, CCR2) and oxidative stress markers (NOX4, SOD3, GPX2); network pharmacology further delineated AGEs-RAGE signaling pathway activation as a key therapeutic mechanism. Serum pharmacochemical analysis identified multiple bioactive compounds in BSTJF, such as catalpol, luteolin, and salidroside, which have known anti-inflammatory and antioxidant effects relevant to PCOS [30, 43–45]. Molecular docking further indicated that additional components, such as corylidin, psoralenoside and isopsoralenoside, may also contribute to the overall effect by potentially binding to targets like RAGE and NOX4. A potential synergistic logic is revealed: corylidin and luteolin showed strong binding to RAGE, while psoralenoside and isopsoralenoside preferentially targeted NOX4. Therefore, the presence of multiple bioactive compounds with complementary mechanisms suggests that the therapeutic efficacy of BSTJF likely arises from its multi-component synergistic effects [46], rather than from a single isolated compound. This potential synergy aligns with the holistic philosophy of TCM and warrants further investigation to quantitatively characterize these interactions.

Our validation in a DHEA-induced PCOS murine model demonstrated BSTJF's therapeutic efficacy in restoring ovarian homeostasis, with comparable effects to the RAGE inhibitor FPS-ZM1 in reducing follicular atresia and inhibiting GC apoptosis. Mechanistically, BSTJF significantly attenuated serum AGEs levels and their intraovarian accumulation while downregulating RAGE expression in GCs, thereby demonstrating that BSTJF mitigates PCOS-associated inflammatory response and GC apoptosis by modulating the AGEs-RAGE signaling axis. This aligns with clinical observations of elevated systemic and intraovarian AGEs concentrations in PCOS women [20, 47]. Pathological AGEs-RAGE axis activation in GCs driven by testosterone could induce endoplasmic reticulum stress in human GC cultures [35]. Intraovarian AGE-RAGE-mediated glycative stress has been implicated as a key driver of PCOS-associated ovarian dysfunction [36]. The co-amplification of AGEs and RAGE in PCOS GCs directly corroborates our findings, confirming aberrant AGEs-RAGE signaling as a conserved pathogenic mechanism. Notably, pharmacological RAGE inhibition has been shown to normalize AGE levels across experimental models [35, 38], while our data further validated BSTJF as a multi-target intervention capable of disrupting this pathogenic axis.

The accumulation of AGEs represents a heterogeneous group of compounds generated through non-enzymatic glycation reactions of sugars and proteins or other biomolecules [48]. Carbonyl stress drives the generation of AGEs and facilitates redox-active cyclization processes that disrupts cellular homeostasis and metabolic networks [49]. Our metabolomic profiling revealed BSTJF’s preferential modulation of carbon/carbohydrate and lipid metabolism pathways in serum, suggesting its potential protection against glycation-induced ovarian dysfunction and PCOS-related glucolipid dysregulation. Furthermore, AGEs are extensively documented as ligands that induce intracellular oxidative stress and inflammatory cascades upon binding to their transmembrane receptor RAGE [24]. These mechanisms align with our observations in PCOS models, which showed systemic oxidant-antioxidant imbalance [16] and pro-/anti-inflammatory dysregulation. Crucially, both BSTJF treatment and RAGE inhibition counteract oxidative stress and inflammation, underscoring their therapeutic potential.

Building upon our prior findings that BSTJF ameliorates PCOS via Sirtuin 3 mediated mitochondrial oxidative stress regulation [16], this study delineates its therapeutic action through NADPH oxidase-dependent redox dysregulation. We demonstrated that BSTJF mitigated oxidative damage (lipid peroxidation, protein oxidation, DNA lesions) and suppressed apoptosis in GCs of PCOS mice. While the pathophysiological role of NADPH oxidase in PCOS remains underexplored, existing evidence links hyperandrogenemia to NOX subunit p47phox upregulation in leukocytes [50], and NOX2 inhibition mitigates ROS overproduction in PCOS ovarian GCs [51]. As a protein complex producing ROS, NOX4 was reported to be highly expressed in PCOS rat ovaries, while NOX4 deficiency inhibited oxidative stress and cell apoptosis in DHEA-induced rat ovaries [29]. Similarly, our data reveal NOX4 overexpression in PCOS GCs, along with exacerbated oxidative cascade and increased GC apoptosis. Both RAGE inhibitor FPS-ZM1 and treatment with BSTJF suppressed NOX4 expression, mechanistically linking the AGEs-RAGE axis to NOX4-driven oxidative stress as previously documented [18].

The MAPK signaling pathway critically regulates NF-κB activation through phosphorylation-dependent IκBα degradation, enabling nuclear translocation of NF-κB p65 to drive transcription of pro-inflammatory mediators (e.g., IL-6, IL-1β and TNF-α) [52]. Our experimental data demonstrated hyperactivation of p38 MAPK phosphorylation and IκBα degradation in ovary of PCOS mice, accompanied by enhanced nuclear translocation of NF-κB p65 in GCs. AGEs-RAGE engagement triggers a redox-inflammatory cascade via NADPH oxidase-mediated ROS overproduction, which activates p38 MAPK and downstream NF-κB [23]. Pharmacological RAGE antagonism via FPS-ZM1 has demonstrated efficacy by suppressing the hyper-phosphorylation of NF-κB p65 and p38 MAPK [38]. Our data suggest that BSTJF inhibits NOX4, as supported by reduced oxidative markers (4-HNE, 3-NT, 8-OHdG). This decrease in NOX4-derived ROS, a key upstream signal, consequently downregulates p38 MAPK phosphorylation. Thus, our study established a causal link between AGEs-RAGE-driven NOX4 activation and subsequent NF-κB nuclear translocation in PCOS GCs. Crucially, unlike FPS-ZM1's singular RAGE antagonism, BSTJF simultaneously inhibited NOX4-driven ROS overproduction and NF-κB-mediated cytokine release, synergistically disrupting oxidative-inflammatory crosstalk in PCOS.

This study advances PCOS therapeutics by elucidating a novel mechanistic axis linking AGEs-RAGE signaling to NOX4-driven oxidative stress and NF-κB-mediated inflammation, through which BSTJF exerts its therapeutic effects. Importantly, this work highlights NOX4 as a tractable node for mitigating both oxidative and inflammatory injury in PCOS, with BSTJF serving as a prototype multi-pathway modulator. Nevertheless, the precise mechanisms underlying the ameliorative effects of BSTJF on ovarian micro-environment in PCOS remain incompletely characterized, particularly the exact cellular targets of its bioactive components. Furthermore, the bioavailable active components of BSTJF require more definitive pharmacological characterization, particularly their direct interactions with targets such as RAGE and NOX4, along with the exact contribution of individual compounds and the possibility of multi-component synergistic effects. Subsequent investigations using compound-specific knockout or selective inhibition approaches would help clarify their specific roles and interactive mechanisms. Another limitation of this study lies in the fact that while our in vivo data strongly suggest the involvement of the AGEs-RAGE/NOX4/NF-κB pathway in the alleviation of oxidative-inflammatory stress by BSTJF in ovarian GCs of PCOS, future studies employing in vitro GC culture models combined with gain- or loss-of-function approaches will be essential to establish direct causal relationships.

## Conclusion

Our study delineates a pathological axis wherein AGEs-RAGE signaling exacerbates PCOS progression. BSTJF alleviates oxidative-inflammatory stress in ovarian GCs of PCOS through AGEs-RAGE/NOX4/NF-κB pathway. These findings provide scientific support for developing Chinese herbal medicine into multi-pathway therapies against PCOS-associated ovarian dysfunction.

## Supplementary Information

Supplementary Material 1.

## Data Availability

The data produced from this study will be made available from the corresponding author on reasonable request.

## Abbreviations

AGEs: Advanced glycation end-products
BSTJF: Bu-Shen-Tian-Jing Formula
DEGs: Differentially expressed genes
DHEA: Dehydroepiandrosterone
FSH: Follicle-stimulating hormone
GCs: Granulosa cells
FSHR: Follicle-stimulating hormone receptor
GO: Gene ontology
GSEA: Gene set enrichment analysis
IPGTT: Intraperitoneal glucose tolerance testing
KEGG: Kyoto encyclopedia of genes and genomes
LC–MS/MS: Liquid chromatography-tandem mass spectrometry
LH: Luteinizing hormone
IL-10: Interleukin-10
IL-1β: Interleukin-1 beta
IL-6: Interleukin-6
NF-κB: Nuclear factor-κB
NOX4: NADPH oxidase isoform 4
PCOS: Polycystic ovary syndrome
PPI: Protein–protein interaction
p38-MAPK: P38 mitogen-activated protein kinase
ROS: Reactive oxygen species
SOD2: Superoxide dismutase 2
TCM: Traditional Chinese medicine
TNF-α: Tumor necrosis factor-alpha
UPLC-Q-TOF MS: Ultra-high performance liquid chromatography coupled with quadrupole time-of-flight mass spectrometry
