# Supplementary material for: Bu-Shen-Tian-Jing Formula alleviates oxidative-inflammatory stress in granulosa cells of polycystic ovary syndrome through AGEs-RAGE/NOX4/NF-κB pathway
Source: Chin Med. 2026 Jan 23;21:50. doi: 10.1186/s13020-026-01333-z (PMC12829069; doi:10.1186/s13020-026-01333-z)
Supplement: Supplementary file 1 — Supplementary Material 1. [file 13020_2026_1333_MOESM1_ESM.docx]

**Supplementary Table 1 Primers used in this study**

| **Gene** | **Primer** | **Sequence (5' - > 3')** |
| --- | --- | --- |
| Human ACTB | Forward | TGGCACCCAGCACAATGAA |
|  | Reverse | CTAAGTCATAGTCCGCCTAGAAGCA |
| Human TNF | Forward | CTGCCTGCTGCACTTTGGAG |
|  | Reverse | ACATGGGCTACAGGCTTGTCACT |
| Human TLR7 | Forward | AAGATGCCTTCCAGTTGCGAT |
|  | Reverse | CCCACCAGACAAACCACACA |
| Human NOX4 | Forward | GGTGGTGGTGCTATTCCTCA |
|  | Reverse | AGCCCTCCTGAAACATGCAA |
| Human CD1D | Forward | GGCCATTCAAGTGCTCAACC |
|  | Reverse | CTCAAGGAGGCCACTGACAA |
| Human GPX2 | Forward | GTGGTCCTTGGCTTCCCTT |
|  | Reverse | GGTGAAGGTGGGCTGGTATC |
| Human CCR2 | Forward | TGCAAATGAGTGGGTCTTTGG |
|  | Reverse | AAACACAGCATGGACAATAGCC |
| Human RAGE | Forward | AACATCACAGCCCGGATTGG |
|  | Reverse | GAAGGAAGAGGGAGCCGTTG |
| Mouse ACTIN | Forward | CATCCGTAAAGACCTCTATGCCAAC |
|  | Reverse | ATGGAGCCACCGATCCACA |
| Mouse IL-6 | Forward | CCACTTCACAAGTCGGAGGCTTA |
|  | Reverse | TGCAAGTGCATCATCGTTGTTC |
| Mouse AKT1 | Forward | CCTTTATTGGCTACAAGGAACGG |
|  | Reverse | CACATGGAAGGTGCGCTCAA |
| Mouse STAT3 | Forward | TCCTGGCACCTTGGATTGAG |
|  | Reverse | TGTGCTGATAGAGGACATTGGA |
| Mouse TNF | Forward | ACTCCAGGCGGTGCCTATGT |
|  | Reverse | GTGAGGGTCTGGGCCATAGAA |
| Mouse EGFR | Forward | AGTTCTGGGTTCGGGAGCA |
|  | Reverse | CACATAGGCTTCGTCAAGGATTTC |
| Mouse BCL2 | Forward | GGTGGTGGAGGAACTCTTCAG |
|  | Reverse | GGTGACATCTCCCTGTTGACG |
| Mouse RAGE | Forward | AGGAGGTCAAGTCCAACTACC |
|  | Reverse | TAGCTTCCCTCAGACACACAT |
| Mouse NOX4 | Forward | TCTCAGGTGTGCATGTAGCC |
|  | Reverse | TCAGACCAGGAATGGTTGTGAA |

| **Metabolites** | **Control** | **PCOS** | **BSTJF-H** | ***P*-values** | | |
| --- | --- | --- | --- | --- | --- | --- |
|  |  |  |  | **PCOS vs. Control** | **BSTJF-H vs. PCOS** | **BSTJF-H vs. Control** |
| L-Gulose (*10^8^) | 6.88 ± 0.71 | 3.25 ± 0.17 | 5.58 ± 0.73 | 0.000 | 0.011 | 0.137 |
| Levoglucosan (*10^7^) | 6.40 ± 0.81 | 0.23 ± 0.48 | 6.37 ± 0.74 | 0.036 | 0.039 | 0.973 |
| D-Allose (*10^7^) | 3.35 ± 0.51 | 1.93 ± 0.27 | 3.29 ± 0.54 | 0.038 | 0.045 | 0.936 |
| Aldehydo-D-Galactose (*10^9^) | 2.67 ± 0.25 | 1.94 ± 0.15 | 2.64 ± 0.27 | 0.035 | 0.042 | 0.926 |
| D-Xylulose 5-phosphate (*10^7^) | 2.77 (2.46, 4.53) | 4.96 (4.71, 5.22) | 3.66 (2.91, 4.44) | 0.036 | 0.021 | 0.294 |
| hreitol (*10^7^) | 1.38 ± 0.16 | 0.90 ± 0.04 | 1.21 ± 0.13 | 0.002 | 0.044 | 0.405 |
| Sedoheptulose (*10^9^) | 7.26 (6.51, 8.00) | 5.41 (5.10, 5.62) | 6.41 (6.02, 7.36) | 0.002 | 0.048 | 0.305 |
| Glucose (*10^8^) | 3.01 (2.03, 3.70) | 1.61 (1.24, 1.83) | 2.08 (1.94, 3.33) | 0.001 | 0.010 | 0.437 |
| Pelargonic acid (*10^7^) | 4.06 ± 0.12 | 3.74 ± 0.08 | 4.24 ± 0.17 | 0.046 | 0.023 | 0.402 |
| (S)-10,16-Dihydroxyhexadecanoic acid (*10^6^) | 5.33 ± 0.67 | 3.70 ± 0.27 | 5.87 ± 0.76 | 0.040 | 0.025 | 0.607 |
| Piperidine acid (*10^7^) | 2.61 ± 0.37 | 6.58 ± 1.12 | 2.29 ± 0.56 | 0.001 | 0.000 | 0.764 |
| Pipecolic acid (*10^7^) | 4.99 ± 0.46 | 2.52 ± 0.26 | 4.28 ± 0.43 | 0.000 | 0.005 | 0.219 |
| Pyrophosphate (*10^7^) | 4.48 ± 0.07 | 5.17 ± 0.22 | 4.33 ± 0.13 | 0.004 | 0.000 | 0.470 |
| Ethyl fumarate (*10^7^) | 1.34 (1.27, 1.42) | 1.51 (1.40, 1.59) | 1.69 (1.65, 1.74) | 0.000 | 0.009 | 0.191 |
| 16-Oxohexadecanoic acid (*10^5^) | 1.99 ± 0.24 | 0.93 ± 0.10 | 1.48 ± 0.17 | 0.000 | 0.042 | 0.054 |

**Supplementary Table 2 Statistical data on the relative levels of the core metabolites between groups**

Note: Data are presented as mean ± SD or median (IQR).

**Supplementary Table 3 Statistical data of KEGG enrichment analysis**

| **Kegg_level_1** | **Kegg_pathway** | **ko_ID** | **Cluster_frequency** | **Metabolome_frequency** | **P-value** |
| --- | --- | --- | --- | --- | --- |
| Organismal Systems | Mineral absorption | ko04978 | 4 out of 36 11.1111111111111% | 13 out of 461 2.81995661605206% | 0.013518814 |
| Human Diseases | Central carbon metabolism in cancer | ko05230 | 5 out of 36 13.8888888888889% | 23 out of 461 4.98915401301518% | 0.026161279 |
| Metabolism | Ascorbate and aldarate metabolism | ko00053 | 3 out of 36 8.33333333333333% | 9 out of 461 1.95227765726681% | 0.026540639 |
| Metabolism | Tryptophan metabolism | ko00380 | 4 out of 36 11.1111111111111% | 17 out of 461 3.68763557483731% | 0.035814308 |
| Organismal Systems | Carbohydrate digestion and absorption | ko04973 | 2 out of 36 5.55555555555556% | 5 out of 461 1.08459869848156% | 0.051081407 |
| Organismal Systems | Protein digestion and absorption | ko04974 | 4 out of 36 11.1111111111111% | 19 out of 461 4.12147505422993% | 0.052057485 |
| Human Diseases | AGE-RAGE signaling pathway in diabetic complications | ko04933 | 1 out of 36 2.77777777777778% | 1 out of 461 0.216919739696312% | 0.078091106 |
| Organismal Systems | Insulin signaling pathway | ko04910 | 1 out of 36 2.77777777777778% | 1 out of 461 0.216919739696312% | 0.078091106 |
| Human Diseases | Non-alcoholic fatty liver disease | ko04932 | 1 out of 36 2.77777777777778% | 1 out of 461 0.216919739696312% | 0.078091106 |
| Environmental Information Processing | Phosphatidylinositol signaling system | ko04070 | 1 out of 36 2.77777777777778% | 1 out of 461 0.216919739696312% | 0.078091106 |
| Human Diseases | Renal cell carcinoma | ko05211 | 1 out of 36 2.77777777777778% | 1 out of 461 0.216919739696312% | 0.078091106 |
| Human Diseases | Shigellosis | ko05131 | 1 out of 36 2.77777777777778% | 1 out of 461 0.216919739696312% | 0.078091106 |
| Metabolism | Biosynthesis of amino acids | ko01230 | 6 out of 36 16.6666666666667% | 41 out of 461 8.89370932754881% | 0.08736058 |
| Metabolism | Biosynthesis of unsaturated fatty acids | ko01040 | 2 out of 36 5.55555555555556% | 7 out of 461 1.51843817787419% | 0.097115596 |
| Metabolism | Pentose phosphate pathway | ko00030 | 2 out of 36 5.55555555555556% | 7 out of 461 1.51843817787419% | 0.097115596 |

Note: This table shows pathways with *P*-value < 0.1.


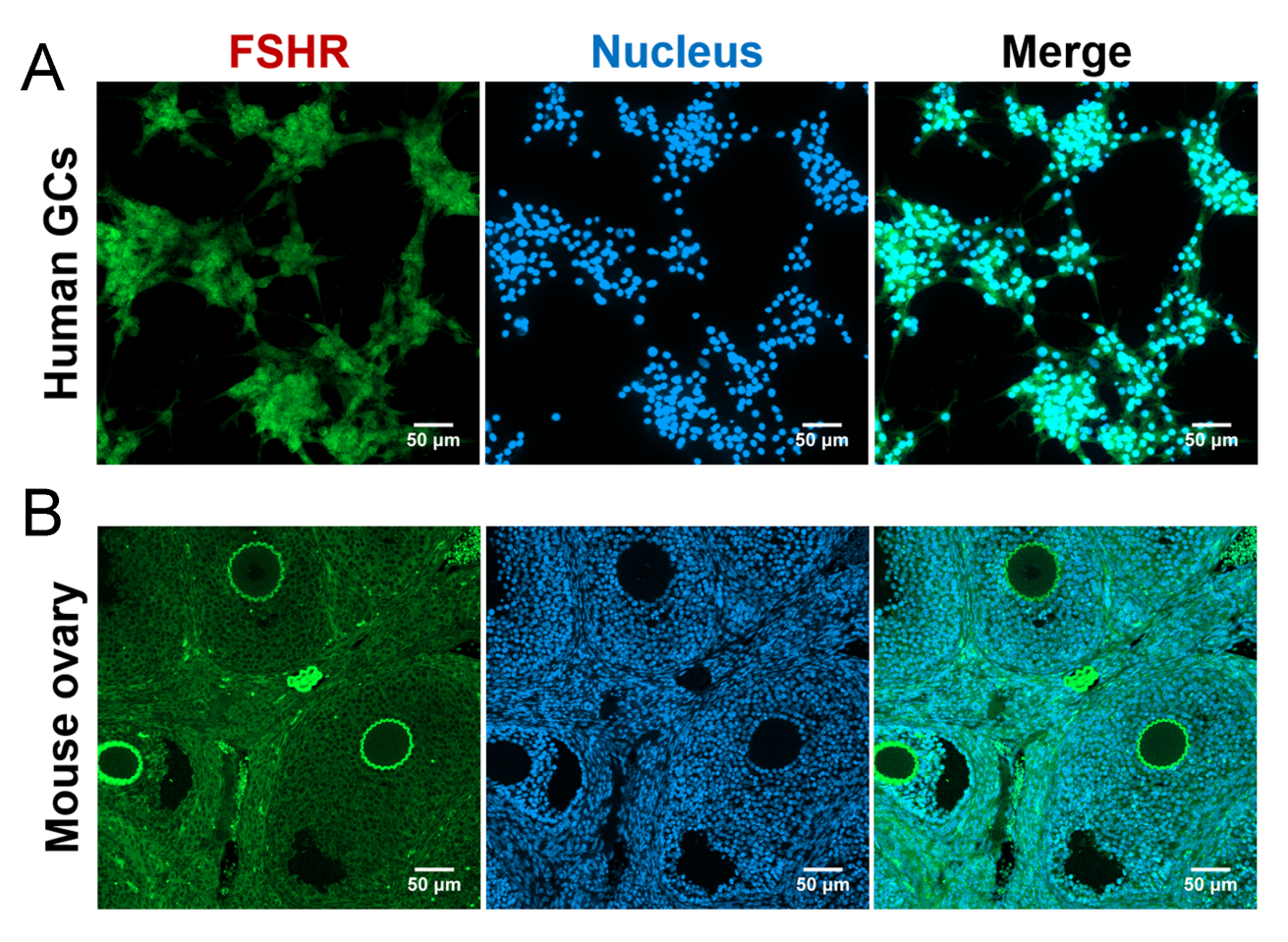
**Supplemental Fig. 1 Identification of ovarian granulosa cells (GCs).** (A) Immunofluorescence labeling of FSHR in human GCs in vitro, showing nuclei stained with DAPI, imaged by confocal microscopy. (B) Immunofluorescence labeling of FSHR in mouse ovarian GCs, showing nuclei stained with DAPI, imaged by confocal microscopy.


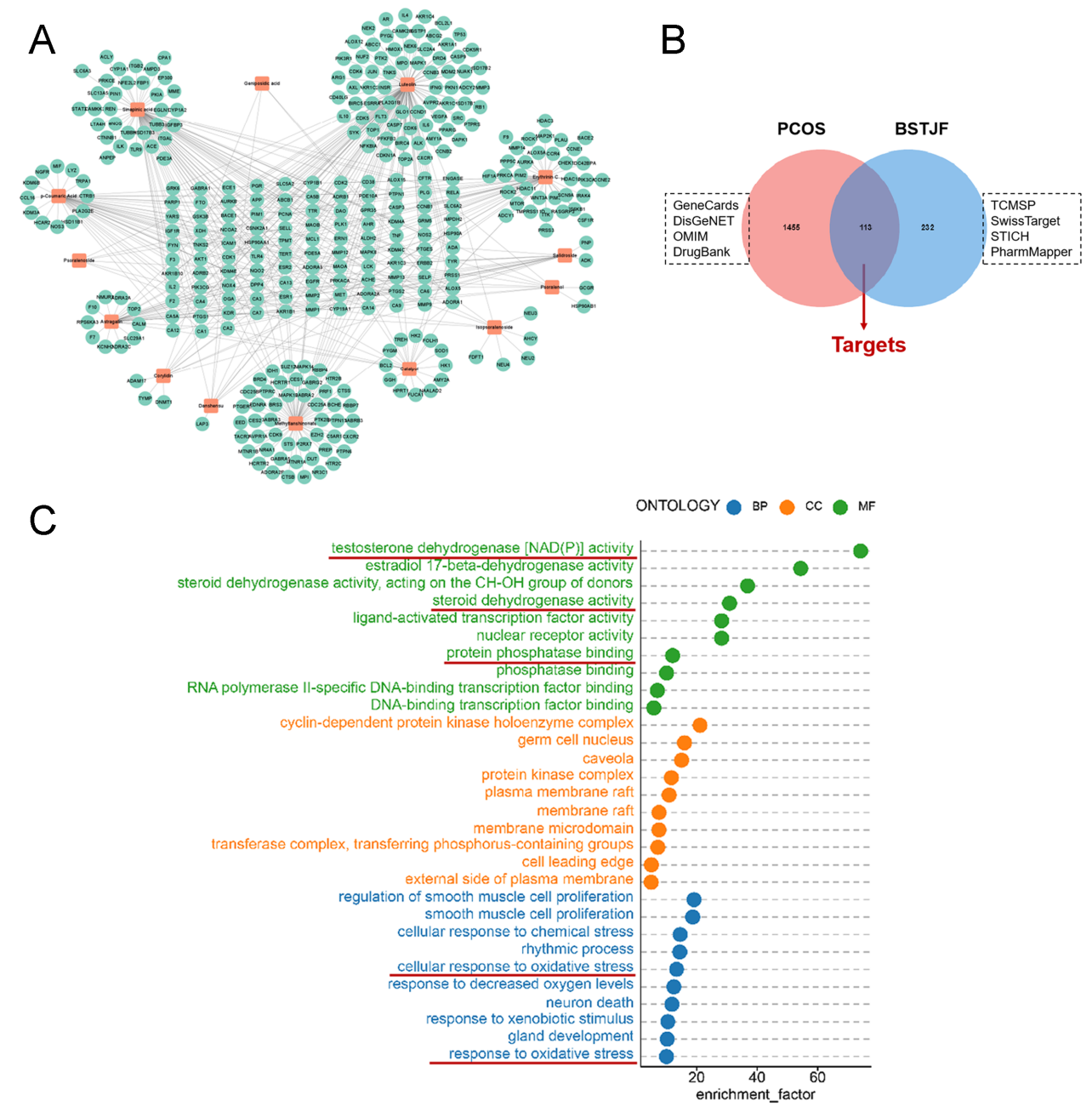


**Supplemental Fig. 2 Network pharmacology of Bushen Tianjing Formula (BSTJF) treating polycystic ovary syndrome (PCOS).** (A) Potential targets of bioactive compounds in BSTJF-containing serum. (B) Venn diagram illustrating the intersection of 113 potential targets of active compounds in BSTJF and PCOS-related genes. (C) Gene Ontology (GO) enrichment analysis of potential therapeutic targets.
